# Validity and reliability of accelerations and orientations measured using wearable sensors during functional activities

**DOI:** 10.1038/s41598-022-18845-x

**Published:** 2022-08-26

**Authors:** Tomasz Cudejko, Kate Button, Mohammad Al-Amri

**Affiliations:** School of Healthcare Sciences, College of Biomedical and Life Sciences, Cardiff University, Cardiff, CF14 4EP UK

**Keywords:** Biomedical engineering, Outcomes research

## Abstract

Wearable sensors may enable the assessment of movement in a real-world setting, but they are not yet a standard practice in the analysis of movement due to the unknown accuracy and reliability with respect to different functional activities. Here, we established the concurrent validity and test–retest reliability of accelerations and orientations measured using affordable novel sensors during squats, jumps, walking and stair ambulation. In this observational study, participants underwent three data collection sessions during one day. Accelerations and orientations from sacrum, thigh and shank were collected using these sensors and already validated gold-standard sensors as the criterion method. We assessed validity by comparing the similarity of signal waveforms with the Linear Fit Method and by comparing mean differences in range values with the Bland–Altman plots. Reliability was assessed by calculating interclass correlation coefficient and standard error of measurements of the range values. Concurrent validity was from fair to excellent in 91% of the cases for accelerations and in 84.4% for orientations. Test–retest reliability of accelerations was from fair to excellent in 97% of cases when the sensors were attached by a researcher, and in 84.4% of cases when the sensors were attached by participants. Test–retest reliability of orientations was from fair to excellent in 88.9% of cases when the sensors were attached by a researcher, and in 68.9% of cases when the sensors were attached by participants. In conclusion, the new affordable sensors provide accurate measures of accelerations and orientations during multiple functional activities in healthy adults. Reliability of the orientations may depend on the ability to replicate the same position of the sensor under test–retest conditions.

## Introduction

For years, the quantitative analysis of human movement has been restricted to gait laboratories that are equipped with many sophisticated measurement devices such as force plates and motion capture systems. However, such equipment is costly, data acquisition and analysis procedures are cumbersome, and the use of such facilities requires space and specialized personnel. Moreover, many people live at a distance from health/research services and continue to be in the workforce with limited time to attend assessment sessions. In addition, the COVID-19 pandemic has had a notable impact on health services which often had to adapt a remote method of service provision. Consequently, there is an unmet need for inexpensive, practical and objective tools enabling monitoring and assessment of human movement in an ambulatory setting^1^.

Wearable sensors, such as Inertial Measurement Units (IMUs), may enable the assessment of movement patterns during functional activities in a real-world setting. IMUs usually contain a 3-axis accelerometer to measure linear accelerations, a 3-axis gyroscope to measure angular velocities, as well as, in some cases, a 3-axis magnetometer to assess earth magnetic field. The fusion of data from multiple IMUs attached to a body segment enables the assessment of free accelerations, orientations and joint kinematics^2^. IMUs are typically lightweight and portable, which facilitate unencumbered movement of a person and do not confine data collection to motion capture systems in the laboratory environment—current gold-standard for movement analysis. Additionally, they are easy to use, cost effective, and can capture data from many movement cycles.

With the rising popularity of IMUs (e.g. Xsens Technologies B.V., Shimmer Sensing, I Measure U, and APDM), there have been an increasing number of research investigating their validity and reliability for movement analysis^3^. The Xsens MVN Analyze system includes wireless motion IMUs (MTw Awinda sensors) and estimates three-dimensional joint kinematics^4^. The reliability and validity of the system for obtaining joint kinematics have been confirmed against gold-standard optoelectronic systems such as the Optotrak system^5,6^, and more recently by our group against the VICON system^7^. Given that size of sensors is important in terms of patients’ acceptability, Xsens has moved toward sensor miniaturization and produced DOT sensors. Given their reduced size, Xsens DOT has become more accessible with regards to capturing movement data anywhere without complex set up that the Xsens MVN system necessitates, potentially opening new avenues for real-world applications. At present however, the validity and reliability of Xsens DOT during functional activities remains unknown.

Current limitation of the IMU validation and reliability research is that it is usually restricted to evaluating joint angles and/or spatio-temporal parameters^3^. These parameters are computed by fusing accelerometer and gyroscope outputs which requires significant processing costs, and the results may be dependent on coding choices. On the other hand, raw IMU outputs such as accelerations and orientations, have been shown to be useful in a wide range of movement-related settings, such as posture recognition^8^, quantification of physical activity levels^9^, determining spatial–temporal gait variables^10^, estimation of hip joint loading patterns^11^, estimation of joint angles^12^, or quantification of knee stability^13^. As such, with recent advances in artificial intelligence methods, machine learning algorithms driven by raw acceleration and orientation signals may provide a unique opportunity to overcome methodological challenges associated with transforming such signals into more complex calculations such as joint kinematic/kinetics, physical activity or posture recognition. To achieve this, the first step would be to test whether IMUs provide accurate and reliable measures of acceleration and orientations, in which research is scarce regarding accelerations, and seems to be non-existing in regards to orientations^3^. Another limitation of the IMUs validity and reliability research is its restriction to assessing usually only walking^3^, which in isolation cannot be extrapolated to a real-world human movement behaviour. Finally, the assumption is the IMUs to be worn and used by people in a real-life setting which presents certain methodological challenges. For example, it is not clear if IMUs can collect accurate and reliable biomechanical data if attached on body parts by people who are not researchers or clinicians.

To the best of our knowledge, there is currently no comprehensive evaluation on the validity and reliability of accelerations and orientations measured with an affordable IMU like the Xsens DOT system during multiple functional activities. Therefore, the first objective of the study was to determine the concurrent validity of accelerations and orientations measured using the Xsens DOT compared to the Xsens MTw Awinda. The second objective was to determine the test–retest reliability of accelerations and orientations measured using the Xsens DOT when attached by a researcher and when attached by a participant. For these objectives, healthy individuals participated in three data collection sessions during one day. Acceleration and orientations from the Xsens DOT and Xsens MTw, were being simultaneously collected while participants performed a series of functional activities. To evaluate validity of the Xsens DOT, we compared acceleration and orientations waveforms obtained from the Xsens DOT and Xsens MTw, using the Linear Fit Method (LFM). We also quantified mean differences in accelerations and orientations range values. To evaluate test–retest reliability of the Xsens DOT when the sensors were attached by a researcher, we compared range values of the acceleration and orientations obtained during the first and second data collection session (sensors attached by the same researcher in both sessions), by using the Interclass Correlation Coefficient (ICC) and the Standard Error of Measurement (SEM). To evaluate test–retest reliability of the Xsens DOT when the sensors were attached by a participant, we compared range values of the acceleration and orientations obtained during the first (sensors attached by a researcher) and third data collection session (sensors attached by participants).

## Results

### Participants’ characteristics

Twenty-one individuals participated in the study. They had a mean age of 30.8 ± 9.0 years, a mean BMI of 25.2 ± 3.9 kg/m^2^, 14 (67%) were male, 7 (33%) were female, and 17 (81%) indicated their right lower-limb as the dominant.

### Concurrent validity

#### Linear fit method

**Figure 1:**
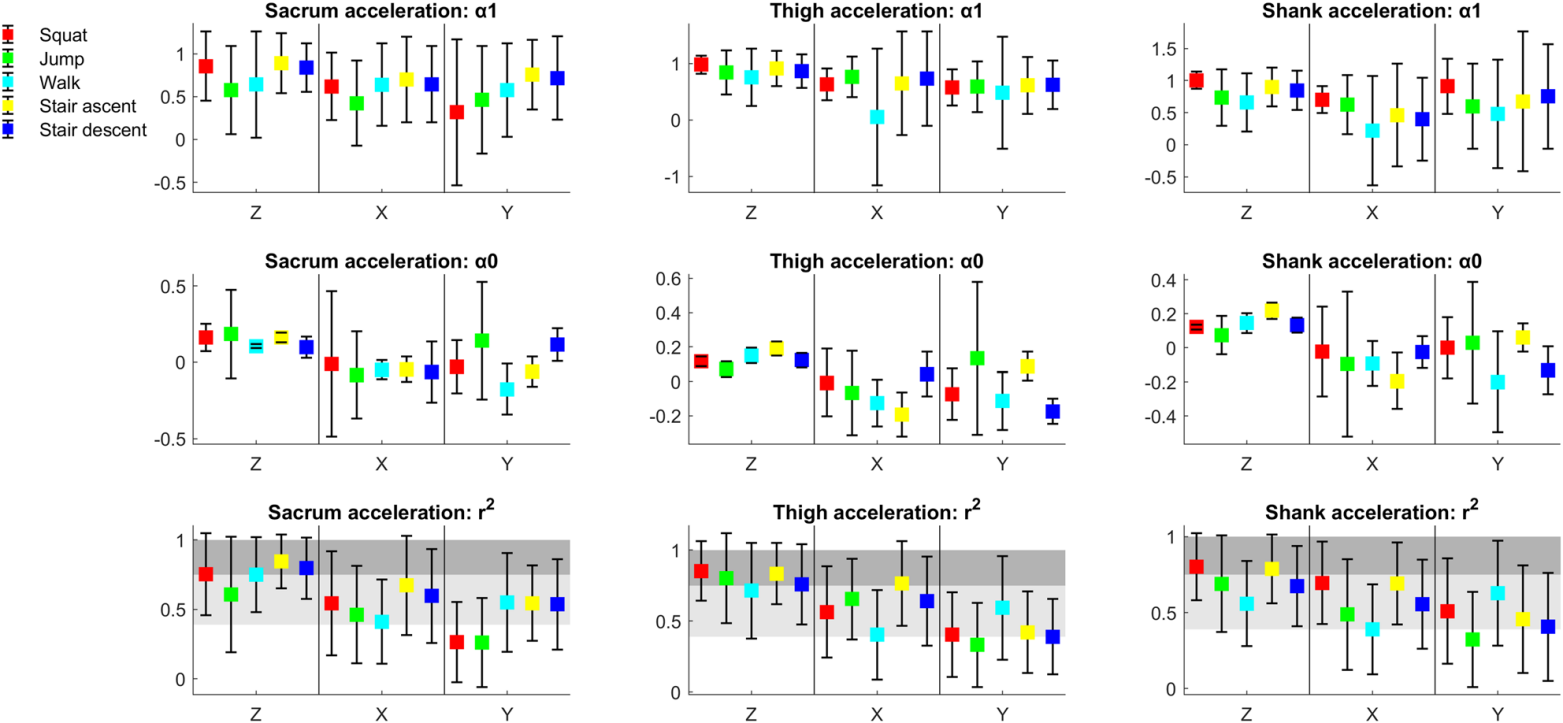
Means (squares) and standard deviations (markers of α1, α0 and r^2^ for individual participants’ acceleration waveforms; light grey shaded areas indicate fair-to-high concurrent validity; dark grey shaded areas indicate excellent concurrent validity; if DOT is identical to MTw then the values of LFM parameters are α1 = 1, α0 = 0, r^2^ = 1, for the full interpretation of the parameter’s values, please see statistical analyses in the methods section.

**Figure 2:**
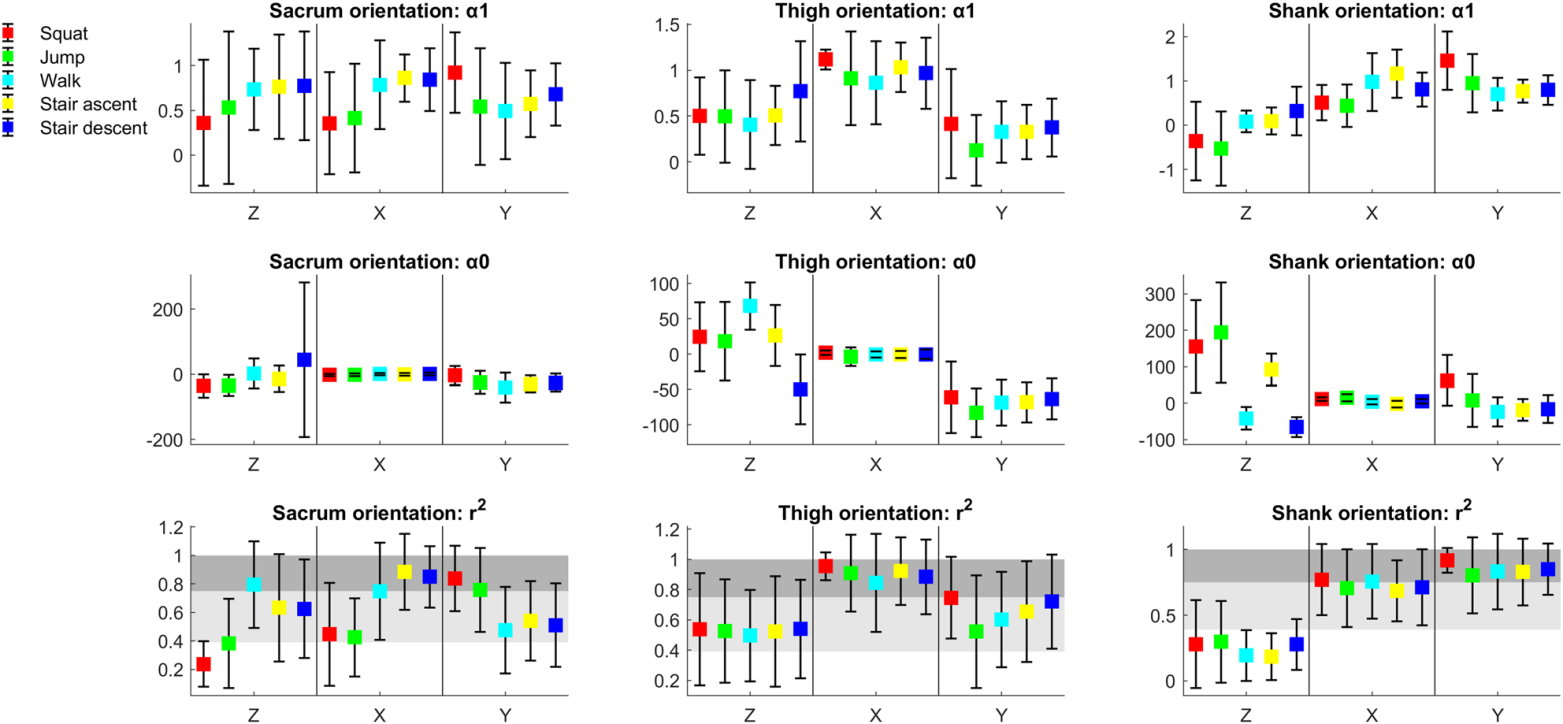
Means (squares) and standard deviations (markers) of α1, α0 and r^2^ for individual participants’’ orientation waveforms; light grey shaded areas indicate fair-to-high concurrent validity; dark grey shaded areas indicate excellent concurrent validity; if DOT is identical to MTw then the values of LFM parameters are α1 = 1, α0 = 0, r^2^ = 1, for the full interpretation of the parameter’s values, please see statistical analyses in the methods section.

r^2^ values for accelerations indicate fair-to-high or excellent concurrent validity for sacrum, shank and thigh sensors for all axes and activities except sacrum sensor in y axis during squats and jumps, and thigh and shank sensor in y axis during jumps (Fig. 1). r^2^ values for orientations indicate fair-to-high or excellent concurrent validity for sacrum, shank and thigh sensors throughout all axes and activities except sacrum sensor in z axis during squats, and shank sensor in z axis during all activities (Fig. 2). Averaged and individual participants’ acceleration and orientation signal waveforms are presented in Supplementary Figs. S1–S10.

#### Mean differences

**Figure 3:**
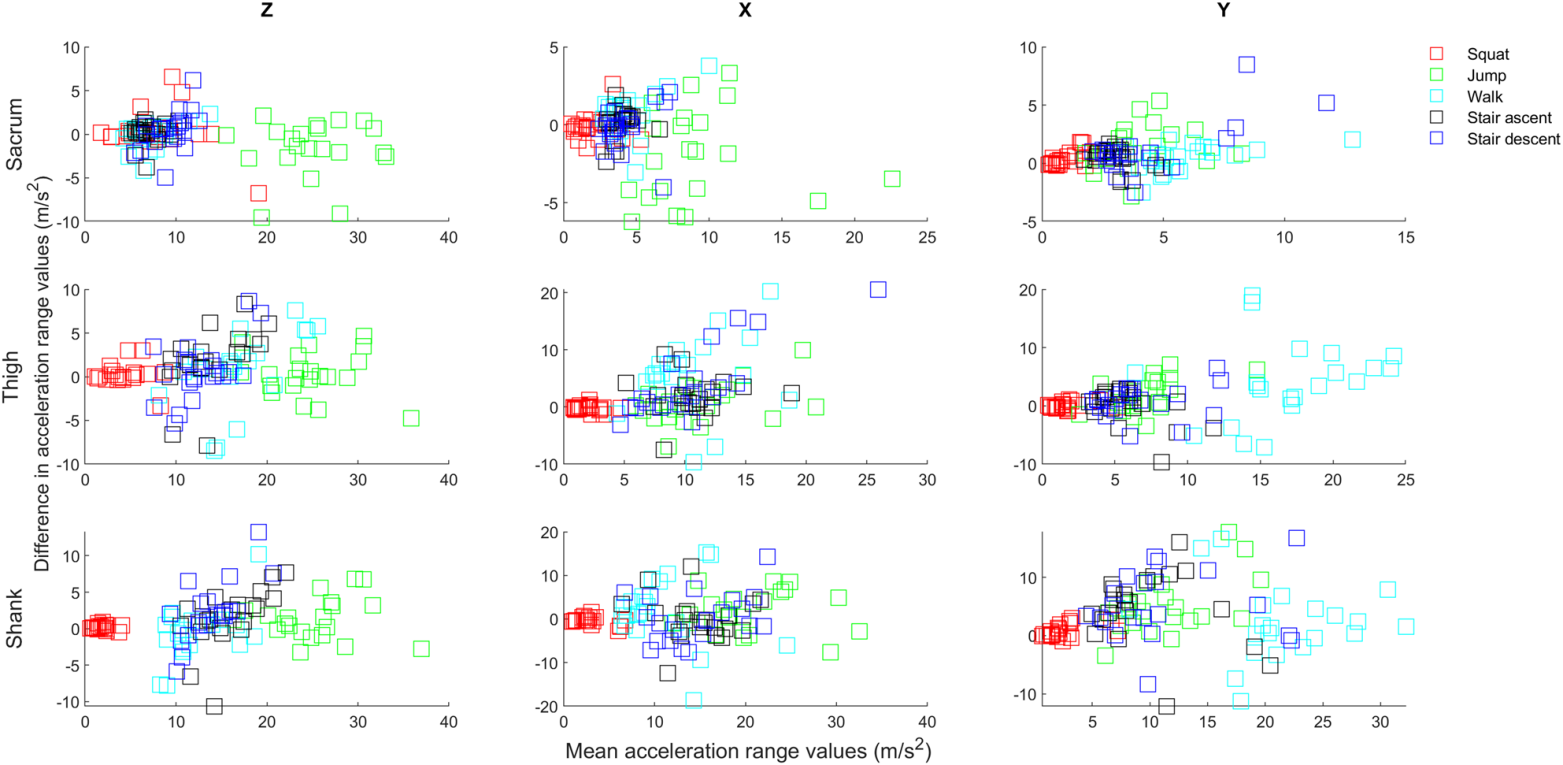
Bland–Altman plots portraying the agreement between the accelerations (combined all activities) collected using the Xsens MTw and Xsens Dot. Y axis—Difference in acceleration range values between the Xsens MTw and Xsens Dot; X axis—Mean acceleration range values of the Xsens MTw and Xsens Dot;

Table 1 presents mean differences [95% CIs] for acceleration range values between Xsens MTw and Xsens Dot. Mean differences in acceleration range values between two sensors ranged from − 1.4 to 2.0 m/s^2^ for sacrum, − 5.6 to 0.2 m/s^2^ for thigh and − 5.2 to 0.5 m/s^2^ for shank. Figure 3 presents Bland–Altman plots of acceleration range values. Supplementary Tables S1–S3 contain means (stds) of the acceleration and orientation range values collected with the Xsens DOT and Xsens MTw during each data collection session.

**Table 1:** Mean differences [95% CIs] for acceleration range values (m/s^2^) between Xsens MTw and Xsens Dot.

Squat			Jump		Walk		Stair ascent		Stair descent	
**Sacrum**										
*z*	− 0.3	[− 5.2, 4.5]	1.6	[− 4.6, 7.7]	0.1	[− 2.9, 2.9]	− 0.2	[− 2.6, 2.3]	− 0.4	[− 4.7, 3.9]
*x*	0.2	[− 1.4, 1.7]	2.0	[− 3.8, 7.7]	− 0.7	[− 3.5, 2.2]	− 0.1	[− 2.0, 2.0]	0.1	[− 2.5, 2.8]
*y*	− 0.4	[− 1.7, 0.8]	− 1.4	[− 5.0, 2.3]	− 0.5	[− 2.7, 1.7]	− 0.4	[− 2.1, 1.4]	− 1.1	[− 5.6,3.2]
Thigh*										
*z*	− 0.2	[− 2.7, 2.2]	− 0.3	[− 5.2, 4.6]	− 0.9	[− 9.2, 7.5]	− 1.9	[− 9.1, 5.3]	− 0.7	[− 7.2, 5.9]
*x*	0.2	[− 0.9, 1.2]	− 0.6	[− 7.4, 6.2]	− 5.6	[− 18.7, 7.5]	− 1.9	[− 8.6, 4.8]	− 4.2	[− 16.7, 8.2]
*y*	− 0.1	[− 1.1, 1.1]	− 2.0	[− 7.6, 3.6]	− 4.1	[− 17.4, 9.1]	0.1	[− 6.0, 6.1]	− 0.8	[− 6.0, 4.5]
Shank*										
*z*	− 0.2	[− 0.8, 0.5]	− 1.3	[− 6.9, 4.3]	0.5	[− 6.6, 7.6]	− 1.4	[− 9.4, 6.6]	− 2.2	[− 10.2, 5.7]
*x*	0.4	[− 1.4, 2.2]	− 1.7	[− 10.9, 7.5]	− 1.9	[− 17.1, 13.3]	− 0.1	[− 9.8, 9.8]	− 0.2	[− 10.9, 10.5]
*y*	− 0.8	[− 2.9, 1.4]	− 5.1	[− 14.6, 4.4]	− 2.1	[− 14.6, 10.3]	− 4.7	[− 16.8, 7.4]	− 5.2	[− 16.7, 6.2]

*CI* confidence interval.

*Data presented for right limb.

**Figure 4:**
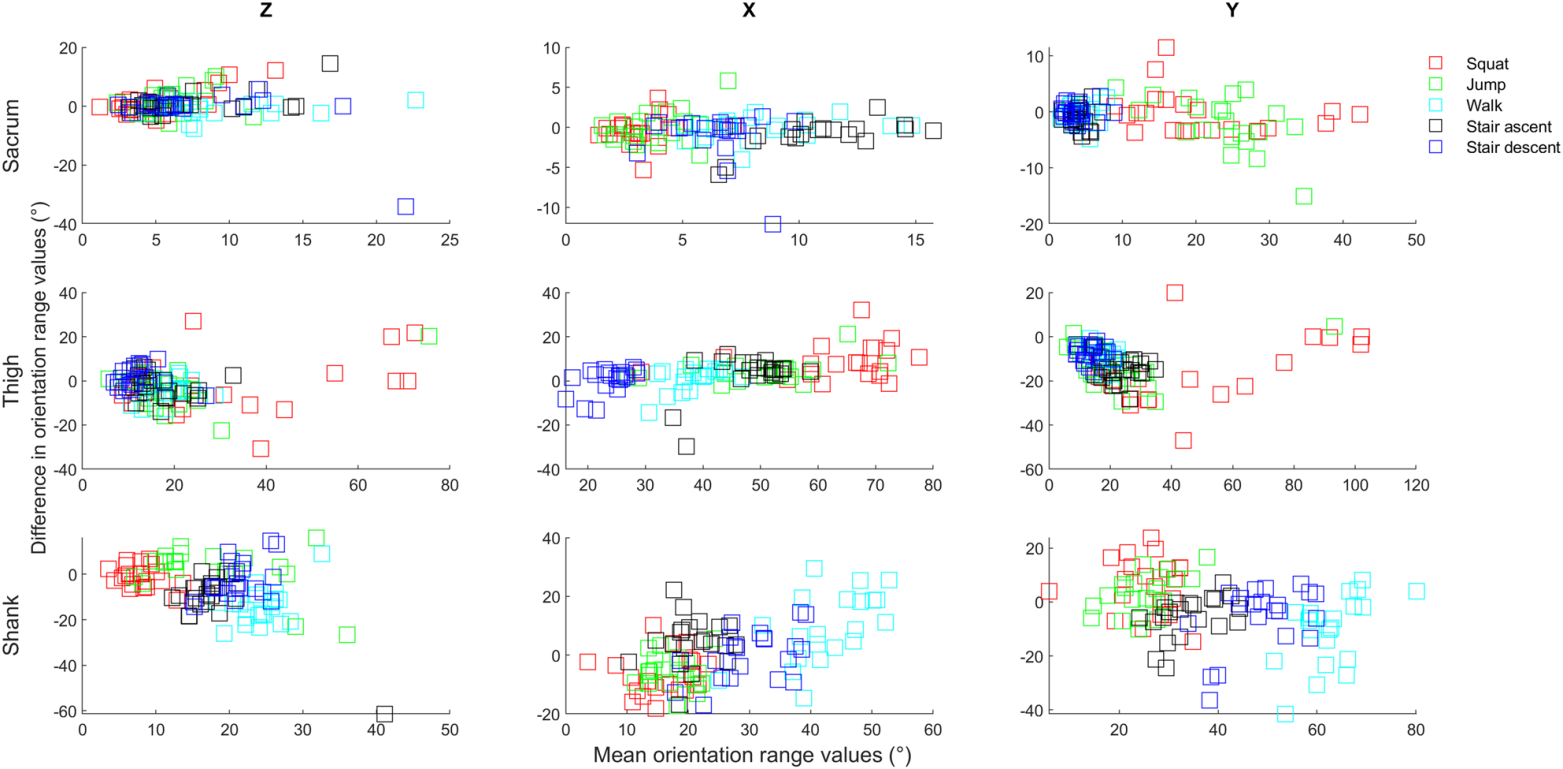
Bland–Altman plots portraying the agreement between the orientations (combined all activities) collected using the Xsens MTw and Xsens Dot. Y axis—Difference in orientation range values between the Xsens MTw and Xsens Dot; X axis—Mean orientation range values the Xsens MTw and Xsens Dot;

Table 2 presents mean differences [95% CIs] for orientation range values between Xsens MTw and Xsens Dot. Mean differences in orientation range values between two sensors ranged from − 1.6 to 1.9° for sacrum, − 8.8 to 16.1° for thigh and − 9.4 to 10.7° for shank. Figure 4 presents Bland–Altman plots of orientation range values.

**Table 2:** Mean differences [95% CIs] for orientation range data between Xsens MTw and Xsens Dot.

Squat			Jump		Walk		Stair ascent		Stair descent	
**Sacrum**										
*z*	− 1.6	[− 10.4, 7.1]	− 1.3	[− 9.3, 6.7]	0.9	[− 3.8, 5.7]	− 0.9	[− 8.3, 6.5]	− 0.7	[− 4.1, 2.7]
*x*	0.4	[− 3.1, 3.8]	0.1	[− 3.8, 4.1]	0.1	[− 2.5, 2.7]	0.9	[− 2.6, 4.3]	1.0	[− 4.7, 6.8]
*y*	0.2	[− 7.3, 7.7]	1.9	[− 7.3, 11.2]	0.4	[− 2.7, 3.6]	1.1	[− 2.4, 4.6]	0.1	[− 2.6, 2.8]
Thigh*										
*z*	1.9	[− 23.8, 27.6]	4.7	[− 8.7, 18.2]	3.7	[− 4.9, 12.3]	3.0	[− 7.4, 13.5]	− 1.5	[− 10.6, 7.4]
*x*	− 8.8	[− 24.2, 6.5]	− 4.3	[− 13.6, 5.0]	− 1.2	[− 11.1, 8.7]	− 3.1	[− 21.4, 15.3]	− 0.4	[− 11.1, 10.3]
*y*	16.1	[− 12.2, 44.4]	13.5	[− 4.6, 31.6]	9.3	[1.5, 17.1]	15.3	[5.0, 25.5]	7.4	[0.4, 14.4]
Shank*										
*z*	0.8	[− 8.3, 10.1]	− 1.9	[− 21.6, 17.7]	14.3	[− 0.7, 29.5]	10.7	[− 14.7, 36.2]	2.7	[− 13.7, 19.1]
*x*	6.7	[− 7.2, 20.7]	5.7	[− 4.9, 16.4]	− 9.4	[− 32.8, 14.0]	− 4.5	[− 20.6, 11.4]	− 0.1	[− 16.8, 16.5]
*y*	− 6.1	[− 26.3, 14.1]	− 2.9	[− 17.4, 11.6]	10.5	[− 14.8, 35.8]	5.5	[− 9.5, 20.6]	5.6	[− 17.5, 28.8]

*CI* confidence interval.

*Data presented for right limb.

### Test-rest reliability (IMUs attached by the researcher)

#### ICCs

**Figure 5:**
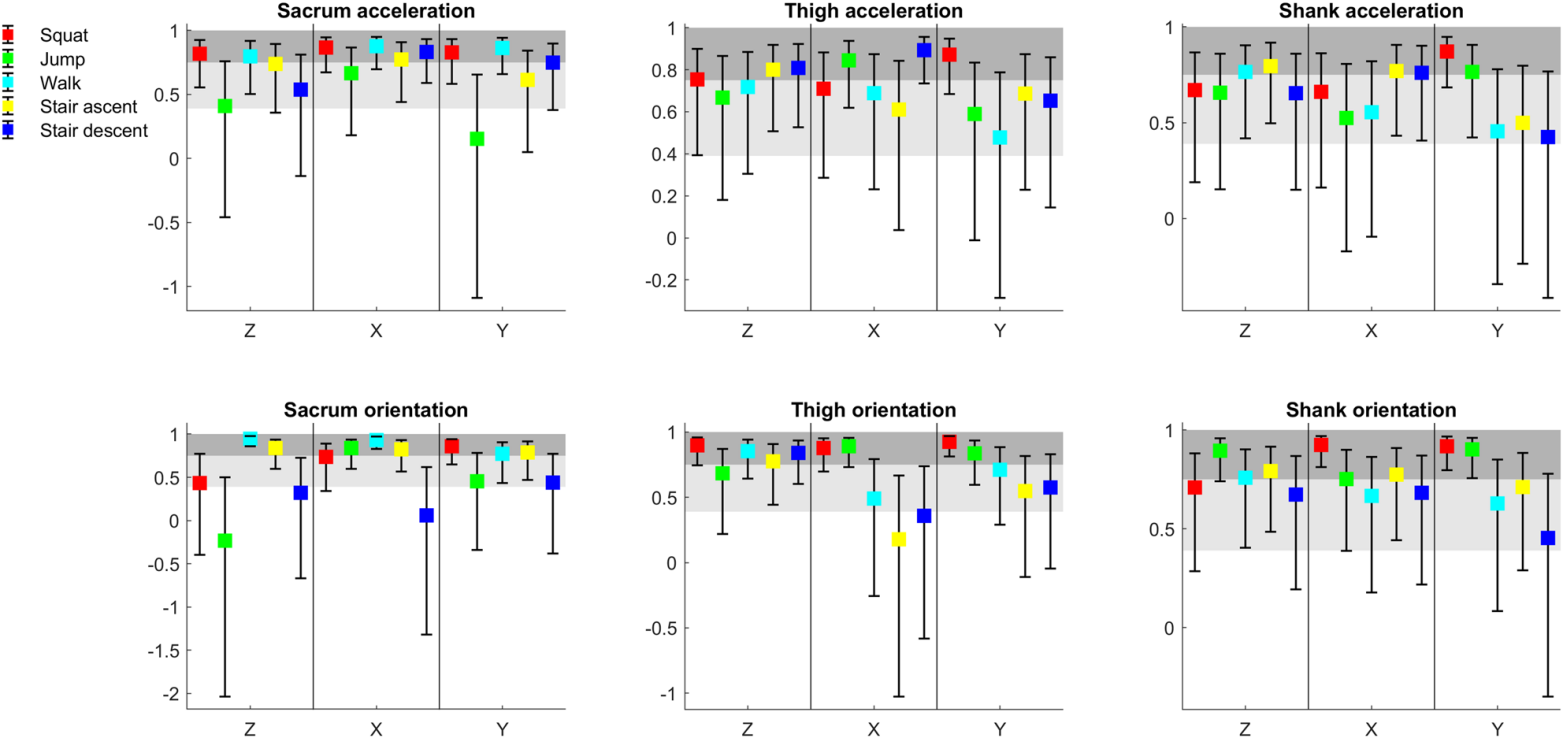
ICCs (95% CI) for acceleration and orientation range values (session 1 vs. session 2); light grey shaded areas indicate fair-to-high test–retest reliability; dark grey shaded areas indicate excellent test–retest reliability.

ICC values for acceleration ranges indicate excellent, fair-to-high or poor test–retest reliability for 46.7%, 51.1% and 2.2% of cases, respectively, when the sensors were placed by the researcher. ICC values for orientations indicate excellent, fair-to-high or poor test–retest reliability for 53.3%, 35.6%, and 11.1% of cases, respectively (Fig. 5).

#### SEMs

**Figure 6:**
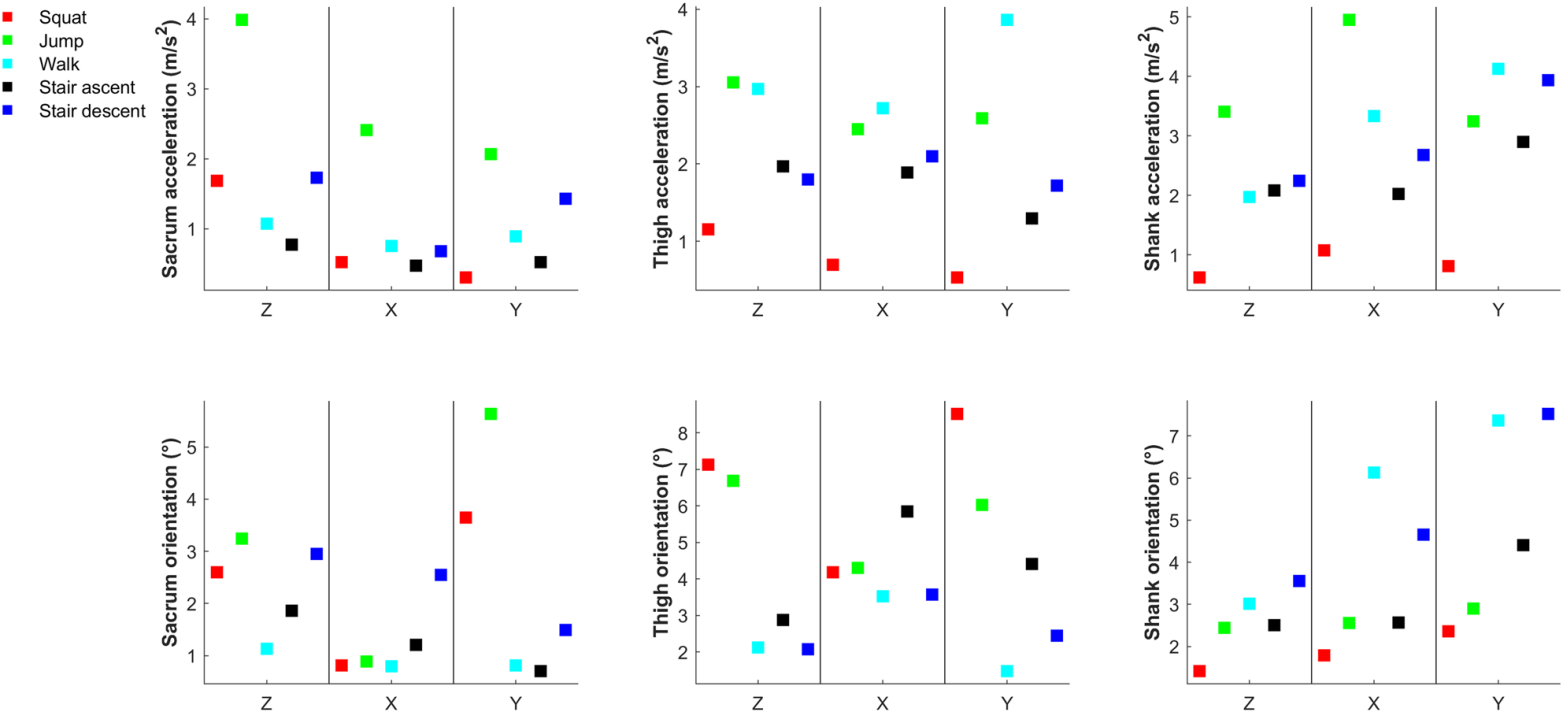
SEMs for acceleration and orientation range values (session 1 vs. session 2).

SEM values were in the range of 0.2 to 5.0 m/s^2^ for accelerations and 0.3 to 7.6° for orientations, except thigh sensor orientation range in y axis during squats (Fig. 6).

### Test-rest reliability (IMUs attached by participants)

#### ICCs

**Figure 7:**
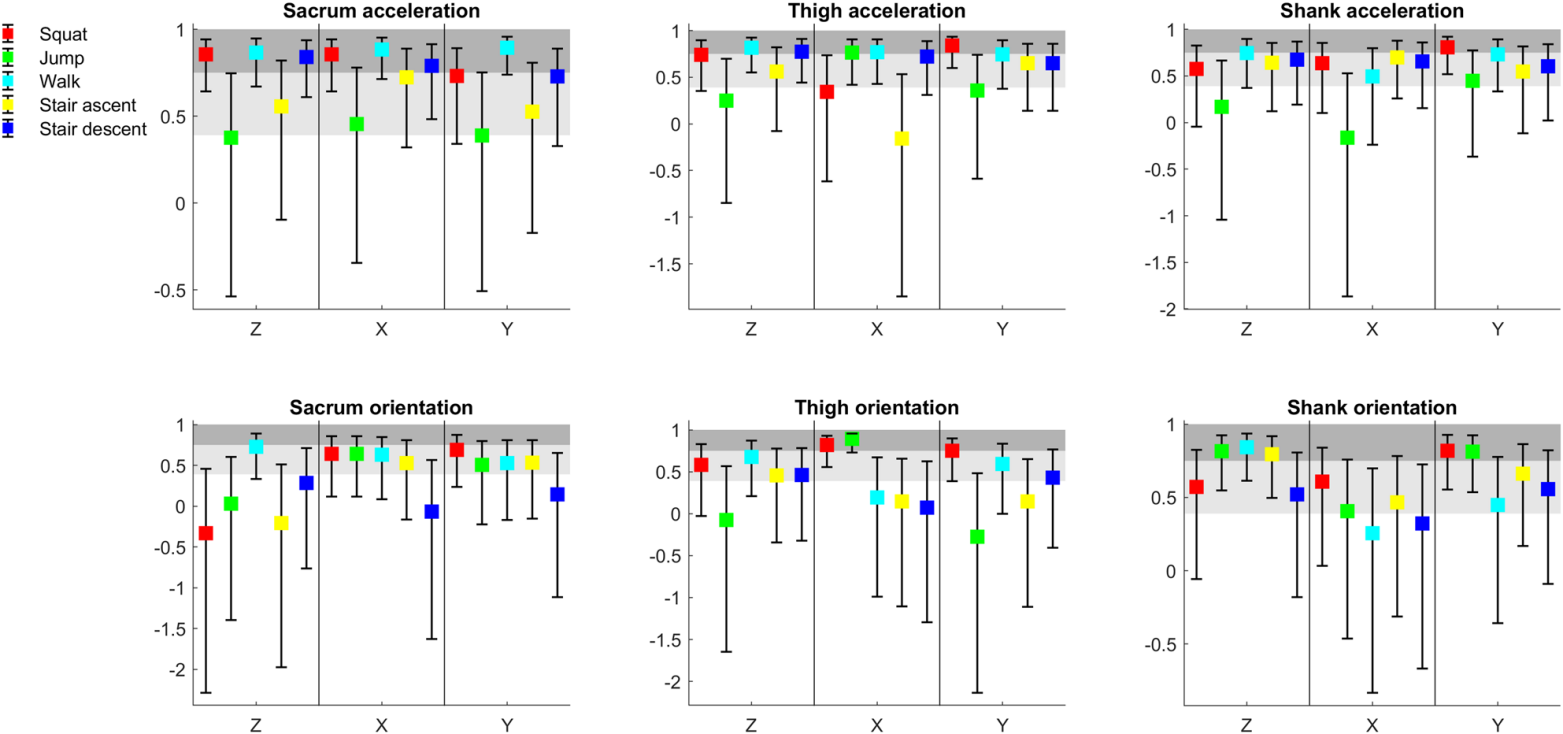
ICCs (95% CI) for acceleration and orientation range values (session 1 vs. session 3); light grey shaded areas indicate fair-to-high test–retest reliability; dark grey shaded areas indicate excellent test–retest reliability.

ICC values for accelerations range values indicate excellent, fair-to-high or poor test–retest reliability for 33.3%, 51.1% and 15.6% of cases when the sensors were attached by participants. ICC values for orientations range indicate excellent, fair-to-high or poor test–retest reliability for 17.8%, 51.1% and 31.1% of cases. (Fig. 7).

#### SEMs

**Figure 8:**
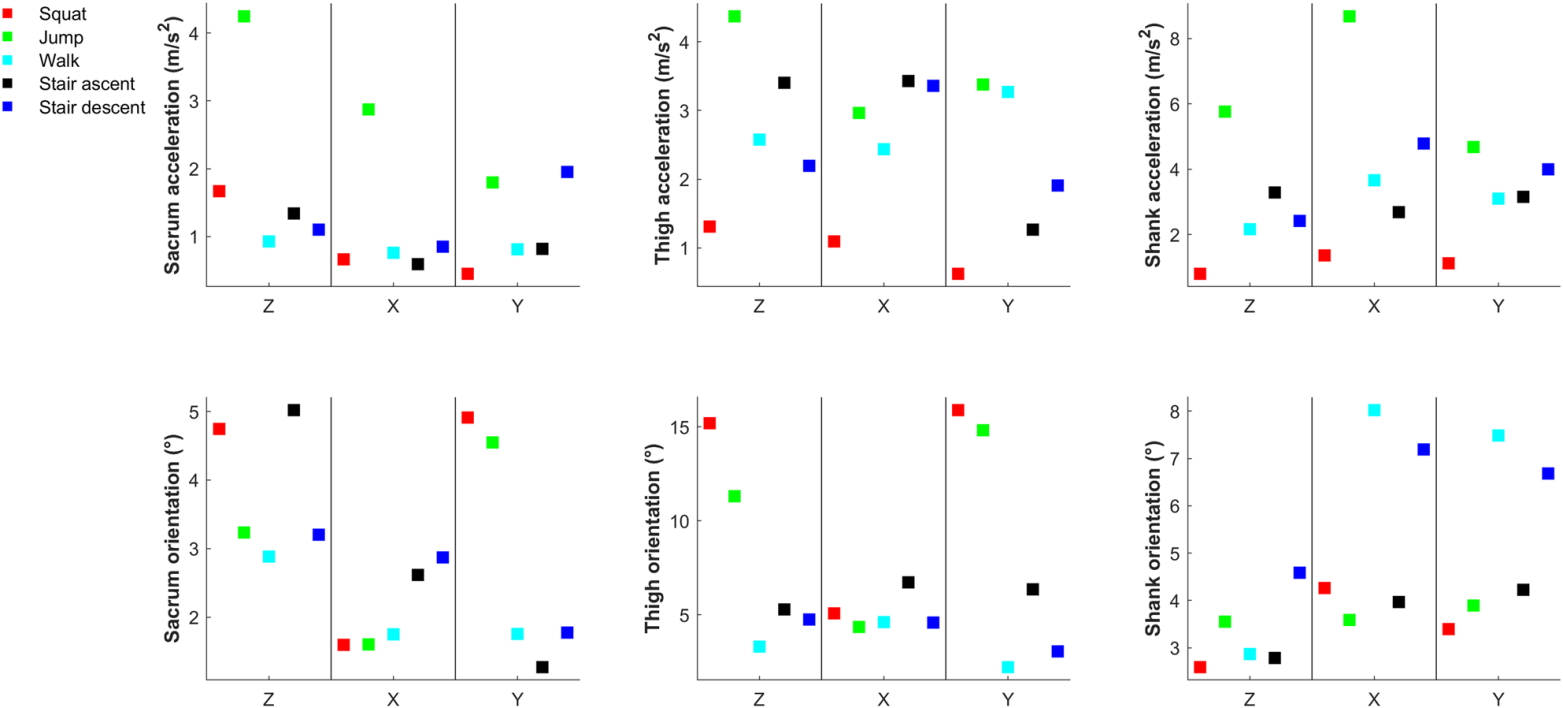
SEMs for acceleration and orientation range values (session 1 vs. session 3).

SEM for accelerations range values were mostly below 5.0 m/s^2^, except shank sensor x axis during jumps. SEM for orientation range values were mostly below 8.0°, except thigh sensor in z and y axes during squats and jumps (Fig. 8).

## Discussion

The objective of the study was to determine the concurrent validity and test–retest reliability of accelerations and orientations measured using Xsens DOT sensor during squats, jumps, walking and stair ambulation.

### Concurrent validity

We demonstrate fair to excellent concurrent validity of accelerations and orientations collected with the Xsens DOT when compared with the Xsens MTw Awinda. This indicates that, the acceleration and orientations waveforms and range values obtained from the Xsens DOT are generally similar to those obtained from the Xsens MTw.

The relatively low similarity in acceleration waveforms in y axis in the jump task (compared to other activities and axes), indicates that the impact that occurs upon initial contact with the ground when landing may contaminate signals. This is confirmed when looking at mean differences in acceleration range values which are higher for thigh and shank compared to the sacrum sensors. These differences may be explained by the difference in the position of the sensors and associated differences in the skin motion artifact and muscle activation patterns during impact loading^14^. We also observed low similarity in waveforms of the z axis orientations of shank sensors in all activities. Differences in the orientation range values are however low, ranging from − 2.2 to 0.5°. The Xsens DOT were attached to the skin by an adhesive tape, on the lateral side of the shank to avoid them falling off during the activities, which is contrary to the Xsens MTw sensors which were attached by a strap. As such, lower similarity of the signal waveforms and higher differences between sensors in orientation and acceleration range values may not necessarily be explained by sensor performance but rather the difference in position of the sensors. Future research comparing IMUs may explore the impact of sensor positions on validation process^15^.

Direct comparisons with other research are difficult due to the large variability in terms of IMUs used, study populations, functional activities, body site locations and statistical methodologies. Kobsar et al.^3^ in their meta-analysis of validity and reliability of IMUs during walking, suggest there is conflicting evidence regarding validity of IMUs for accelerations measured from IMUs placed on the trunk, whereas data on orientations is not present. As such, our results extend and significantly contribute to the body of research by including both accelerations and orientations during multiple functional activities, as well as a more challenging dynamic task; the vertical jump.

These findings have certain practical implications. First, Xsens DOT is more lightweight and flexible to use, and less expensive than Xsens MTw, and as such, provides more flexibility for clinical use for both a patient and a clinician. In light of these findings, Xsens DOT may potentially be used for measuring physical activity levels, falls, gait stability and so forth. Integration of accelerometer and orientations signals, may enable more accurate estimation of these metrics which are traditionally obtained through a bias-prone patient reported outcomes such as questionnaires. Second, despite their widespread use in biomechanical research, calibrating accelerometer and orientation output to joint kinematics, physical activity levels or posture recognition, presents significant methodological challenges. Differences in biomechanics across populations, which are specific to age and/or clinical disease present, mandates that algorithms to delineate these parameters from accelerometer and orientation output be specifically developed for particular populations. Pattern recognition methodologies, such as those utilizing machine learning approaches, have the potential to significantly reduce the processing pipeline and improve the accuracy of accelerometer and orientation-based assessments of joint kinematics, physical activity levels, and/or activity recognition. Although this is not yet in place clinically, research shows that artificial intelligence algorithms can reliably predict certain biomechanical parameters during functional activities without specific knowledge regarding the position of the IMU relative to the attached segment or the body characteristics of the wearer^16,17^.

### Test–retest reliability

Second, we show mostly fair to excellent test–retest reliability of accelerations, independent of who attached the sensors (researcher vs researcher or researcher vs participant). Although direct comparisons with previous literature are difficult due to previously mentioned methodological differences between studies, current findings seem to be in accordance with previous literature. Lyytinen et al.^18^ demonstrate fair to excellent intra-rater reliability of accelerations obtained from a shank-mounted accelerometer during walking and stair ambulation in healthy adults. Similarly, Kavanagh et al.^19^ observed fair to excellent intra-rater and inter-rater reliability of sacrum and shank-mounted accelerations during level walking in heathy male adults. Our findings extend these body of research, and are important from a practical perspective. As long as the person attaching the sensors on body locations has knowledge in anatomy (e.g., a clinician) or has detailed instructions provided (e.g., a patient), the same person does not need to place the sensors for each testing session to obtain reliable acceleration readings. Nevertheless, we observed that seven out of 45 acceleration outcomes had ICCs indicating poor test–retest reliability when the IMUs were attached by participants, compared to only one outcome when the IMUs were attached by the same researcher. We notice that participants tended to perform the activities with a higher velocity during the last session (when the IMUs were attached by participants) compared to the first session (when the IMUs were attached by the researcher) (Supplementary Tables S1–S3). The likely explanation behind this can be a learning effect, and/or the fact that participants had only five Xsens DOT IMUs attached during the last session, compared to the first session where they were additionally equipped with 17 Xsens MTw sensors, surface electromyography and foot pressure insoles (data not presented here, see data collection in the methods).

**Figure 9:**
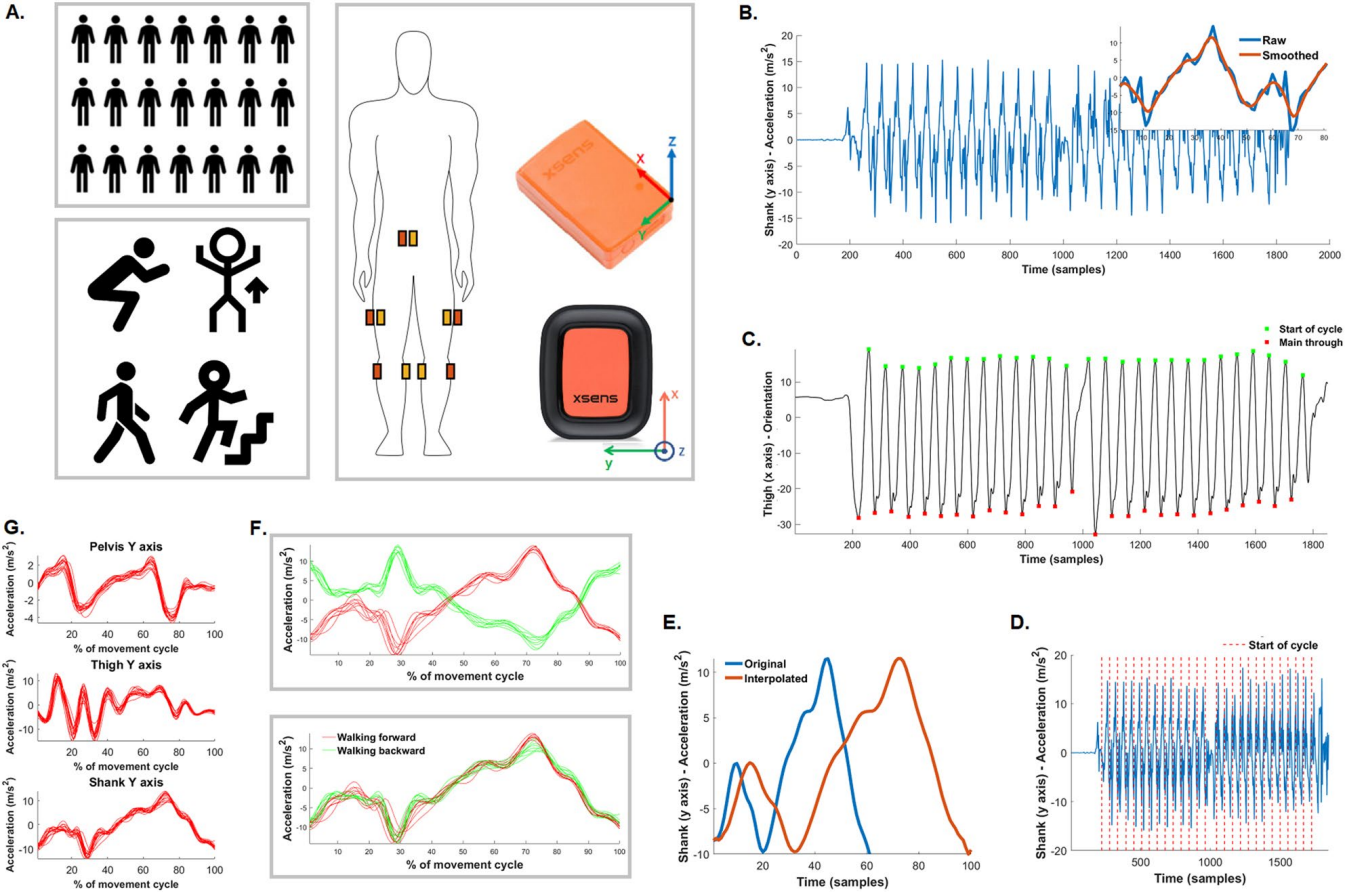
A methodological framework for data collection (**A**) and data processing (**B**–**G**) using a random participant’s Xsens DOT data during a walking trial. (**A**) Number of participants; body site location for IMUs placements within sacrum and lower limbs during the first and second data collection session; Xsens MTw (top sensor) and Xsens DOT (bottom sensor) IMUs with their sensor coordinate systems; functional activities during all data collections (squat, jump, walking, stair ambulation); (**B**) participants’ performed during all data collection sessions; (**B**) Data extraction and effects of Butterworth filter (shank acceleration data in y axis from a middle of a walking trial); (**C**) Identification of start of movement cycles (thigh orientation data in x axis); (**D**) Segmentation of signals; (**E**) Signal interpolation; (**F**) An example of inversion for acceleration signal during walking trial; (**G**) Extraction of all movement cycles data per outcome.

Finally, we observed that test–retest reliability of orientations was mostly from fair to excellent when the sensors were attached by a researcher, however ranged from poor to excellent when the sensors were attached by participants. We observed lower ICC for orientations ranges for sacrum and thigh compared to the shank. The observation that the shank had the highest repeatability is perhaps not surprising given that the lower extremity segments are strictly coordinated during locomotion to provide a precise trajectory for the foot. Also, in terms of repeatability of sensors placement, it is easier to locate appropriate placements for shank given the presence of reference points such as patella, tibial tuberosity or fibula head rather than for thigh and sacrum. Participants could not see where exactly they were attaching the sensor over the sacrum. Also, DOT sensors were placed posteriorly in relation to the MTw sensors in the first data collection session (Fig. 9A). In the third session however, we did not use the MTw sensors so the DOT they were placed centrally, resulting in the different placement location compared to the first session. As such, the sensor positioned over the sacrum may have been tilted in a wide variety of ways due to the inaccuracy in positioning, and changes in the position associated with postural alignment or lumbar lordosis. Together these limitations have the potential to compromise the quality of orientation data recorded from body-mounted sensors during human movement, especially under test–retest conditions. This is an also an important finding from a practical perspective, because it means that orientation data should be treated cautiously when the sensors are attached by different persons i.e., a researcher vs a participant.

We acknowledge that evaluating test–retest reliability of accelerations in each axis has its limitations. Although we have instructed participants to perform the activities in the same manner throughout all data collection session, it was not possible to ensure the repeatability of the motion trajectory in each axis. To validate our results, we have quantified the Euclidean norm of acceleration, which takes into account all the axes together^20^. The ICCs and SEMs for the Euclidean norm of acceleration range values for sacrum, thigh and shank during all activities are presented in the Supplementary Fig. S11. We observed that ICC values for the Euclidean norm of acceleration range values indicated excellent or fair-to-high test–retest reliability in six and nine out of 15 cases, respectively when the sensors were attached by the researcher. When the sensors were attached by participants, we observed ICC values to be excellent, fair-to-high or poor in six, six and three out of 15 cases, respectively. SEM for the Euclidean norm of acceleration range values were below 5.7 m/s^2^ independent who attached the sensors, except the shank sensor during jumps when this was attached by participants.

### Strengths and limitations

First, contrary to previous validity and reliability IMU research, we did not restrict our study to only walking assessment, but investigated multiple functional activities, providing first insights regarding potential performance of the IMUs in real-world movement behaviour. The second strength of the study, is a comprehensive statistical evaluation, where we provide both absolute and relative metrics of validity and reliability. For example, a waveform validity statistic takes into account the time-series evolution of the signal, and therefore provides more comprehensive approach for examining validity of 3D accelerations and orientation instead of the mean signal amplitude computed over several movement cycles. Finally, to our knowledge, we are the first to evaluate the performance of the IMUs when these are attached on body site locations by a non-researcher and/or non-clinician.

This study has also some limitations. First, participants were healthy adults who attended a single testing session. Further research is needed to assess the validity and test–retest reliability of the Xsens DOT in clinical and older populations over a multi-day testing session. Second, although standardized, there might have been certain limitations regarding the instructions we provided to participants about how to attach the IMUs on body parts. These should have been first tested on members of the public and adjusted accordingly based on the feedback received. Third, a researcher corrected participants in case they incorrectly attached sensors i.e., up-side down or in a wrong body part. Although it occurred with several participants only, the effects of this on the results is unclear. Next, although participants had 10 min break between data collection sessions, sensors occasionally left a mark on participant’s skin after being removed. We consider the potential impact of these to be minimal; however, it remains possible that the skin marks influenced the attachment of the sensors in the subsequent sessions, and thus influenced the test–retest reliability. Moreover, participants were also equipped with surface electromyography and wireless in-shoe foot pressure insoles during the first and second data collection. This could have constrained their movement compared to the third session, resulting in a difference in accelerations and orientations not explained by sensor performance Due to methodological constrains, we did not randomize the order in which the IMUs were attached. Future research should consider randomizing the order of the attachment of IMUs between researcher and participants. Finally, although we collected data from both right and left lower extremity, we decided to presented data only for the right limb. We do not expect the results to differ between the limbs, as these were healthy participants with no reported or visually observed conditions that would result in a lower-limb movement pattern difference. We conducted a paired samples t-test (normally distributed data) comparing estimated load between right and left foot obtained from instrumented pressure insoles at the time of the data collection described in this study. The results showed there are no significant differences between limbs in this metric, independent on the activity and data collection session (Supplementary Table S4), suggesting that acceleration and orientation values between limbs were likely similar during the first two sessions.

## Methods

### Study design

This was an observational study. All assessments were conducted during one day at the School of Healthcare Sciences at Cardiff University, in the period between September 2021 and December 2021. Reporting adheres to the Guidelines for Reporting Reliability and Agreement Studies^21^. The School of Healthcare Sciences Research Ethics Committee of the Cardiff University (REC791; 24th May 2021) approved the study.

### Participants

We recruited healthy participants aged ≥ 18 years old from the university community via adverts in university intranet, with no known neurological, cardiovascular, or musculoskeletal conditions that would affect movement. Written informed consent was obtained from each participant prior to participation in the study.

### Sample size calculation

The required sample size (*n* = 16) was determined according to the recommendation for estimating sample size for reliability studies, using α = 0.05, β = 0.2, *n* = 3, *p*0 = 0.4 and *p*1 = 0.7, where α is level of significance, β is the type II error, *n* is number of data collection sessions*, p*0 is the minimally acceptable level of reliability and *p*1 is the expected level of reliability^22^.

### IMUs

We collected free accelerations and orientations using two models of IMUs i.e., Xsens DOT and Xsens MTw Awinda (Xsens Technologies B.V, Enschede, The Netherlands) (Table 3; Fig. 9A). Xsens MTw Awinda has been used as the criterion system given its established validity against optoelectronic motion capture systems^5–7^.

**Table 3:** Technical specification of the Xsens DOT and the Xsens MTw.

Specifications	Xsens Dot	Xsens MTw
Dimensions	36.3 × 30.3 × 10.8	47.0 × 30.0 × 13.0
Weight	11.2 g	16 g
Sampling frequency	60 Hz	60 Hz
Ranges	± 2000°/s, ± 16 g, ± 8 G	± 2000°/s, ± 160 m/s^2^, ± 1.9 G,

Dimensions: length x width x height (mm); Ranges are for: gyroscope, accelerometer, and magnetometer; G- Gauss.

All raw sensor readings (accelerations, angular velocity, earth magnetic field) are in the right-handed Cartesian coordinate system, which is body-fixed to the device and defined as the sensor co-ordinate system (Fig. 1A). These are then fitted into the Xsens Kalman Filter Core and down-sampled at 60 Hz through a protocol called strap-down integration to compute 3-D free accelerations (in m/s^2^) and 3-D orientations (in degrees). 3D free acceleration (acceleration subtracted by the gravity component) and 3D orientations from Xsens DOT and MTw are accelerations and orientations of the sensor coordinate system with respect to the local earth coordinate system, defined as a right-handed Cartesian coordinate system with: X positive to the East, Y positive to the North, and Z positive when pointing up^23,24^.

### Data collection

Each participant underwent three data collection sessions during one day. At the start, we took anthropometric measurements including weight, height, and body segments’ lengths. Five Xsens DOT sensors were then placed on lower limbs and sacrum by a researcher (TC) (Fig. 9A). Following this, 17 Xsens MTw sensors were placed in accordance with Xsens instructions^24^ on the lower extremities and sacrum by TC and on the trunk and upper extremities by another researcher (JW). The DOT sensors were held in position with medical grade double-sided adhesive tape. The MTw sensors were secured using elasticated Velcro straps or were mounted on the Xsens suit. Each sensor was secured with adhesive one-sided medical tape to minimize any movement. The data presented in this manuscript is part of a larger study which collected data from surface electromyography (EMG) and wireless in-shoe pressure insoles. The data from these systems were being collected during the first and the second data collection session. The EMG sensors were placed on the right and left lower-extremity on the following muscles: rectus femoris, semitendinosus, biceps femoris, tibialis anterior, gastrocnemius lateralis and gastrocnemius medialis. The placement of the EMG sensors and the in-shoe pressure insoles did not affect the placement of the DOT and MTw sensors.

Prior to data collection, the MTw sensors were calibrated within the MVN Analyze Pro software (version 2022.01) by asking participants to perform a single level walk and stand in an N-pose (standing still with upper limbs along the waistline). During this process the software establishes the relation between segment and sensor orientations. The Xsens DOT App (version 2021.0) on Apple iPhone 12 (software version 15.0) was used to start and stop the DOT sensors, to assign the sensors to the specific body site location and record data. Each participant performed the following activities while data from each sensor were simultaneously being recorded: double leg squats (eight repetitions), vertical jumps (eight repetitions), level walking (twice 15 m, continuously in a corridor) and stair ambulation (one floor level four times) (Fig. 9A). Prior to performing each activity, the participant was provided a demonstration by the researchers, and was allowed to ask any questions. All activities were performed by participants at their comfortable approach and a self-selected speed.

Once all activities were completed, we removed all lower body sensors. The participant rested for around 10 min, and then the DOT and MTw sensors were placed by the same researcher (TC). The upper body MTw sensors were not removed during this rest period. The participant then repeated the activities in the same order as during the first session. Once all activities were completed, we removed all body sensors. The participant rested for around 10 min, and then placed by himself/herself five DOT sensors on their lower limbs and sacrum according to written instructions provided by the researchers. The instructions were standardized and included images and written text. Images depicted body site’s locations where each sensor needed to be attached. The text further specified how to identify the body site’s locations in reference to other easily identifiable anatomical landmarks, and instructed participants on the right orientation the sensors needed to be attached. In case of any doubts regarding the instructions, participants were encouraged to seek clarifications with the researchers. Following the attachment of all sensors, participants then repeated the activities in the same order as during the second session. We instructed participants to perform the activities in the same manner throughout all data collection sessions. If we visually determined the activity was performed differently (for example with significantly higher velocity), the trial was repeated.

### Data processing

All data processing and statistical analyses were carried out in MATLAB (R2020b, The MathWorks Inc., Natick, MA, USA). Data collected using the MTw sensors were exported as an *.mvnx file and then transformed into a *.mat file. Data collected using the DOT sensors were exported as an *.csv file and then transformed into a *.mat file. Orientations from the DOT and MTw sensors were collected in Quaternions, thus, for the ease of interpretation, we transformed orientations into Euler angles using the ‘quat2angle’ MATLAB function with the ‘zxy’ rotation sequence. All data were filtered with a 6-Hz low-pass second-order Butterworth filter (Fig. 9B). For the comparison purposes, we extracted data per movement cycle. For all activities, the start of the movement cycle was defined as local maxima in thigh orientation *x* axis that preceded each local minima (main through) in the signal, whereas the end of the movement cycle was defined as the subsequent local maxima that succeeded each local minima (main through) in thigh orientation x axis (Fig. 9C). After identifying the indices that defined start of the movement cycle, we segmented all the signals (Fig. 9D). For the data analysis purposes, we discarded the first and the last movement cycle. Because movement cycles differed in length, for the comparison purposes between and within participants, the cycles needed to be rescaled to 100%. To identify data values at 1% intervals we interpolated cycles to 101 (0–100%) data points (Fig. 9E). Sacrum, thigh and shank accelerations in x and y axes were inverted (for walking back), to account for change of direction during walking trials (Fig. 9F). All outcomes were segmented based on the identified movement cycles and extracted for statistical analyses (Fig. 9G). We divided stair ambulation into stair ascents and stair descents. We quantified range of accelerations and orientations per movement cycle, defined as minimum value subtracted from maximum value. These were calculated for each participant as an average of all movement cycles, before being averaged across all participants. No other post-processing was performed on the data provided by the two sensors.

### Statistical analyses

#### Concurrent validity

To evaluate concurrent validity, we compared data obtained from the DOT and the MTw sensors collected during the first data collection session. First, the Xsens DOT and the MTw sensors were compared by evaluating the acceleration and orientations waveforms using the linear fit method (LFM). The LFM relies on the interpretation of the values of three parameters: α1 (mean variation of DOT waveform for every one unit change in MTw waveform), α0 (shift i.e. value of DOT waveform when MTw waveform is equal to 0), and r^2^ (square of the Pearson’s correlation coefficient *R*; strength of the linear relationship between waveforms)^25^. As such, if DOT is identical to MTw then the values of LFM parameters are α1 = 1, α0 = 0, r^2^ = 1. Here, we mainly base our interpretation on r^2^ where r^2^ ≥ 0.75 indicates excellent concurrent validity, 0.4–0.74 fair to good, and ≤ 0.39 poor. According to Cohen^26^, in social sciences r^2^ value ≤ 0.12 indicate poor, 0.13–0.25 values indicate acceptable, and ≥ 0.26 indicate excellent validity. It is generally expected that r^2^ value be higher in technical sciences, hence we believe our criteria are valid. In addition, we evaluated concurrent validity by calculating differences in range of accelerations and orientations across the movement cycle by using Bland–Altman plots^27^. Validity was evaluated for each activity (squat, jump, walk, stair ascent, and stair descent), each body part (sacrum, right thigh, and right shank), and each sensor axis (z,x,y).

#### Test–retest reliability

To evaluate test–retest reliability of the Xsens DOT when the sensors were attached by the researcher, we compared acceleration and orientation ranges from first and the second data collection sessions. To evaluate test–retest reliability when the sensors were attached by the researcher and participants, we compared data from the first and the third data collection sessions. Reliability was quantified using the Intraclass Correlation Coefficient (ICC) with the two-way random effects model (consistency), and the Standard Error of Measurement (SEM), for the acceleration and orientations range values. ICC reflects the relative reliability, which is the degree to which two or more sets of measures are maintained over repeated measurements^28^. ICC ≥ 0.75 indicates excellent reliability, ICC 0.4–0.74 indicates fair-to-high reliability, and ICC ≤ 0.39 indicates poor repeatability^29^. The SEM was quantified using the following equation.

$$SEM=SD x \sqrt{1}-ICC$$

SD refers to the standard deviation of the mean values of ranges from the two IMUs. SEM was used to evaluate absolute reliability and provides information on variability over repeated measurements^28^. We used SEM for descriptive purposes mainly, as, to our knowledge, reference values for acceleration and orientations do not exist. Reliability was evaluated for each activity, each body part and each sensor axis.

## Supplementary Information

Supplementary Information 1.

Supplementary Information 2.

## Data Availability

Range values of accelerations and orientations for all activities and each participant are included in this published article [and its supplementary information files]. The raw dataset analysed during the current study is available from the corresponding author on reasonable request.
