# Supplementary material for: Validity and reliability of accelerations and orientations measured using wearable sensors during functional activities
Source: Sci Rep. 2022 Aug 26;12:14619. doi: 10.1038/s41598-022-18845-x (PMC9417076; doi:10.1038/s41598-022-18845-x)
Supplement: Supplementary file 1 — Supplementary Information 1. [file 41598_2022_18845_MOESM1_ESM.pdf]

**Title**

Validity and reliability of accelerations and orientations measured using wearable sensors during functional activities.

**Authors**

Tomasz Cudejko[1], Kate Button[1], and Mohammad Al-Amri[1]

**Affiliations**

[1] School of Healthcare Sciences, College of Biomedical and Life Sciences, Cardiff University, Cardiff, CF14 4EP, United Kingdom

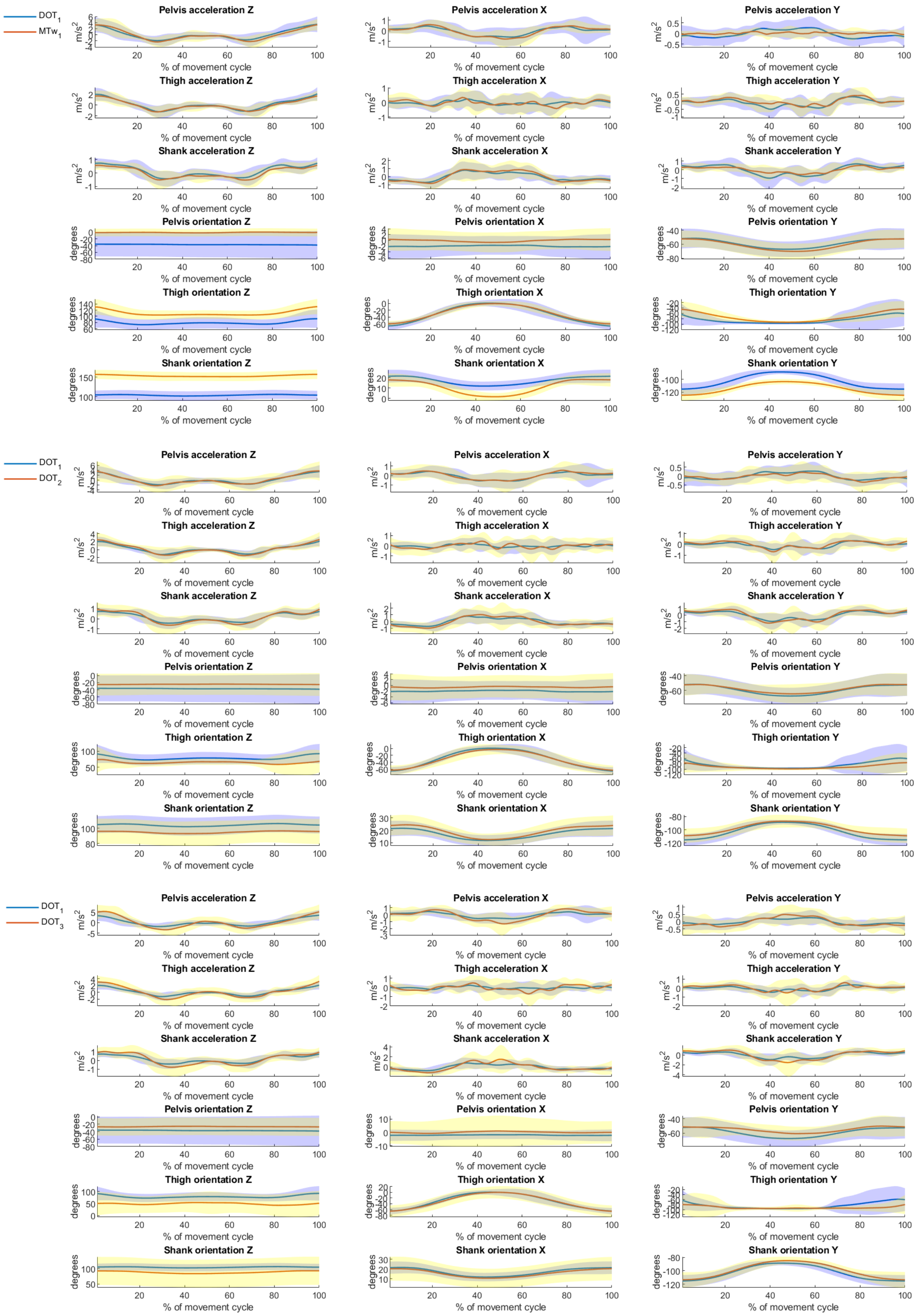


**Supplementary Figure 1.** Mean (stds) waveforms of accelerations and orientations across all participants during squats

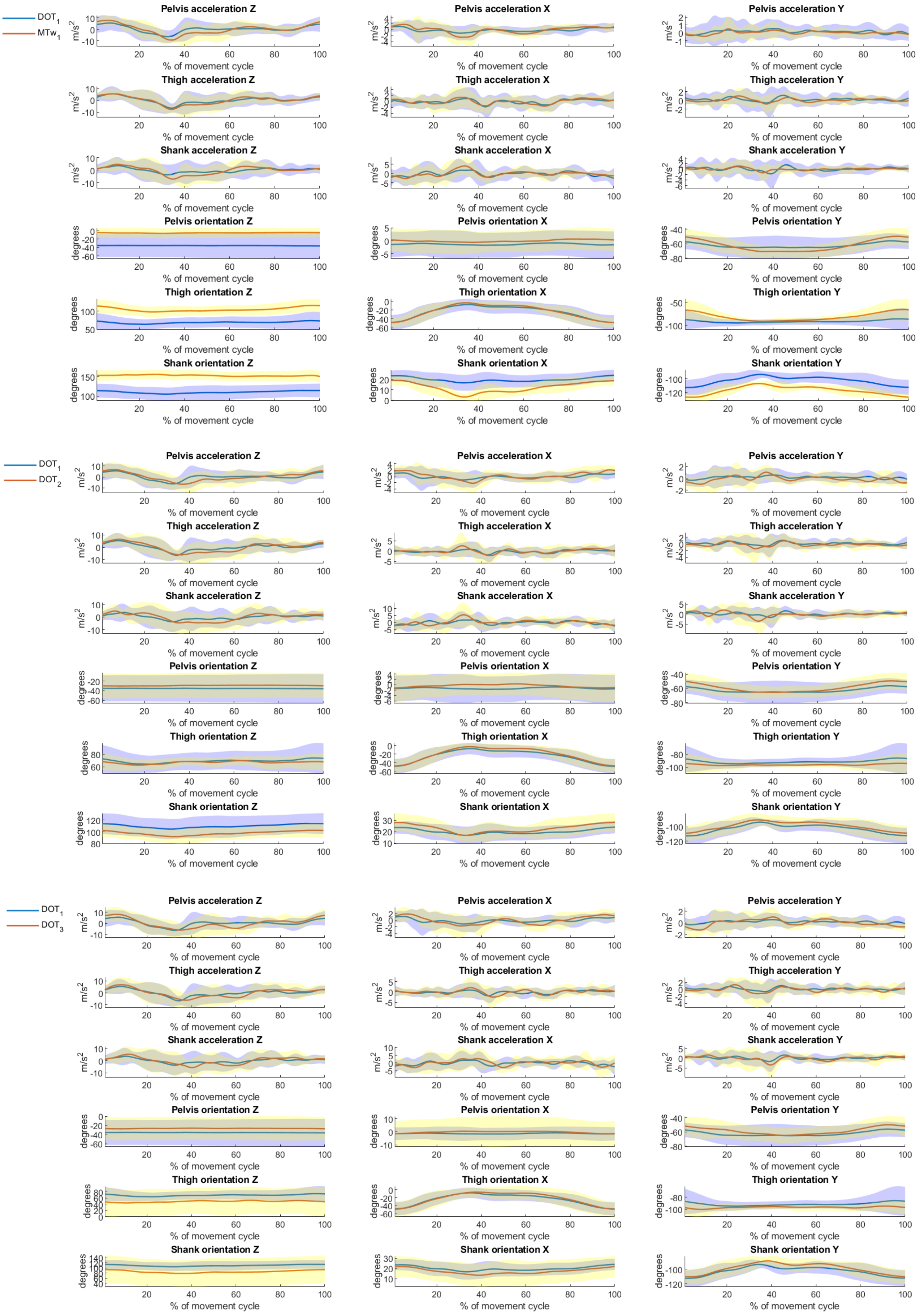


**Supplementary Figure 2.**. Mean (stds) waveforms of accelerations and orientations across all participants during jumps

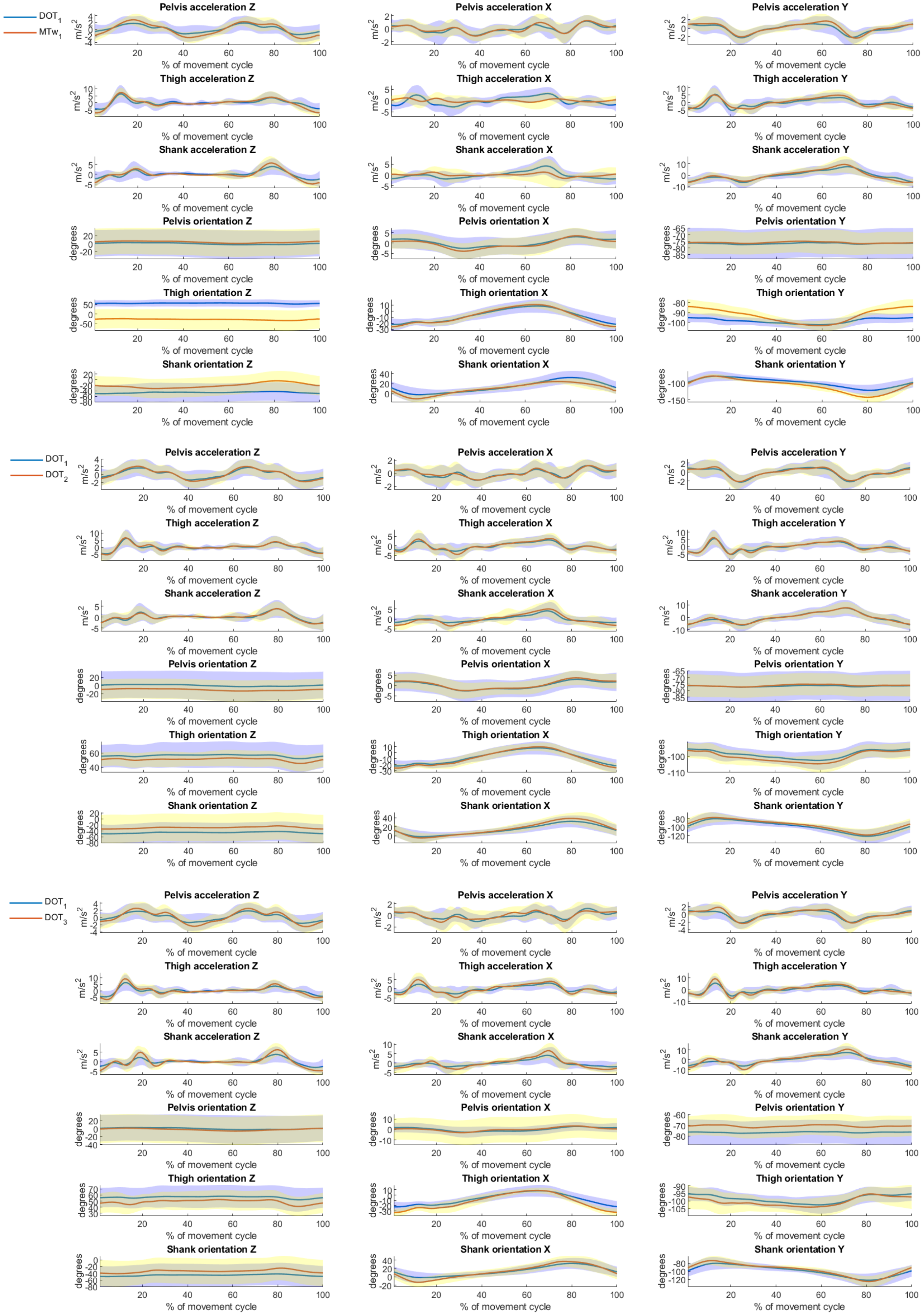


**Supplementary Figure 3**. Mean (stds) waveforms of accelerations and orientations across all participants during walking

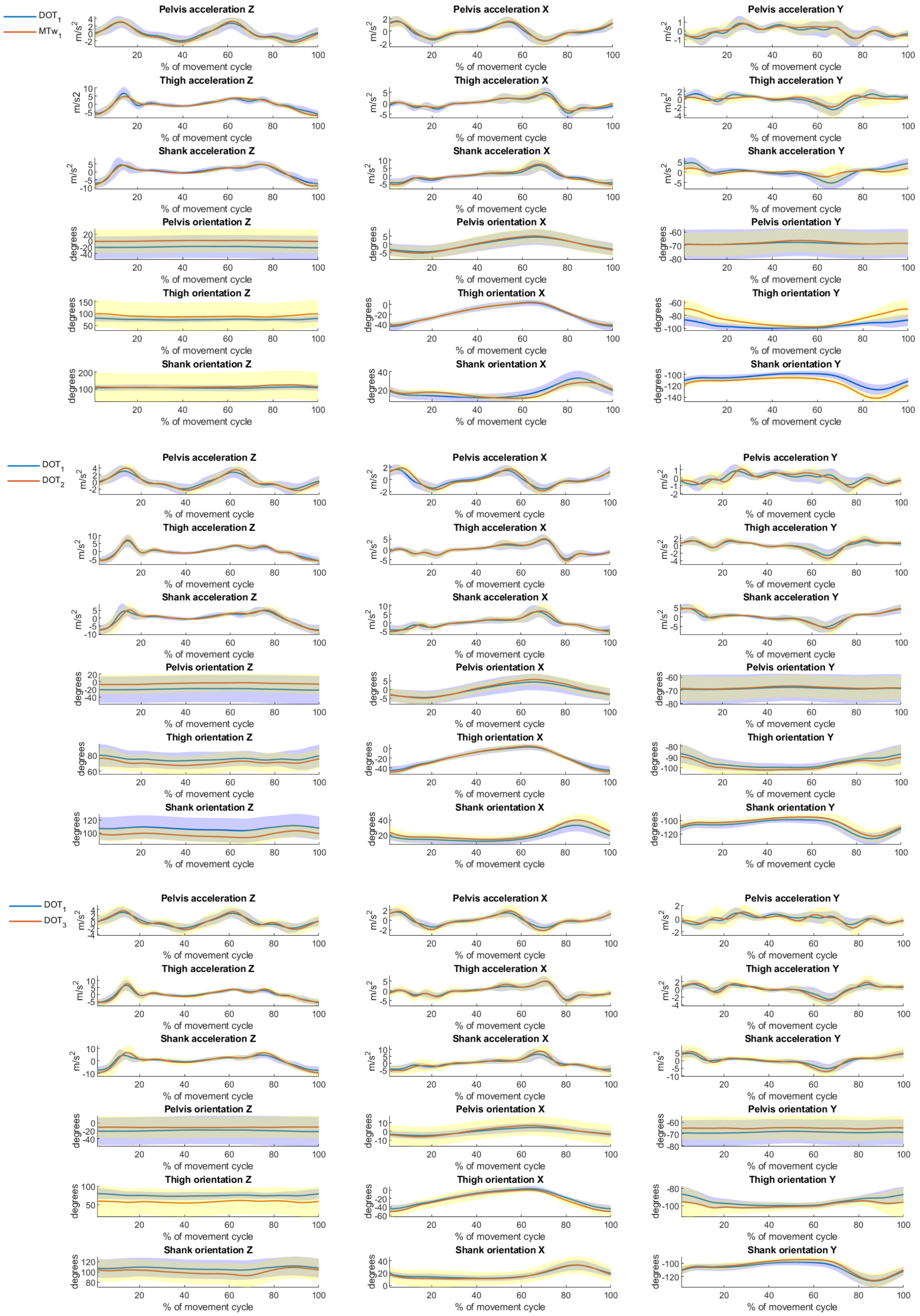


**Supplementary Figure 4.** Mean (stds) waveforms of accelerations and orientations across all participants during stair ascents

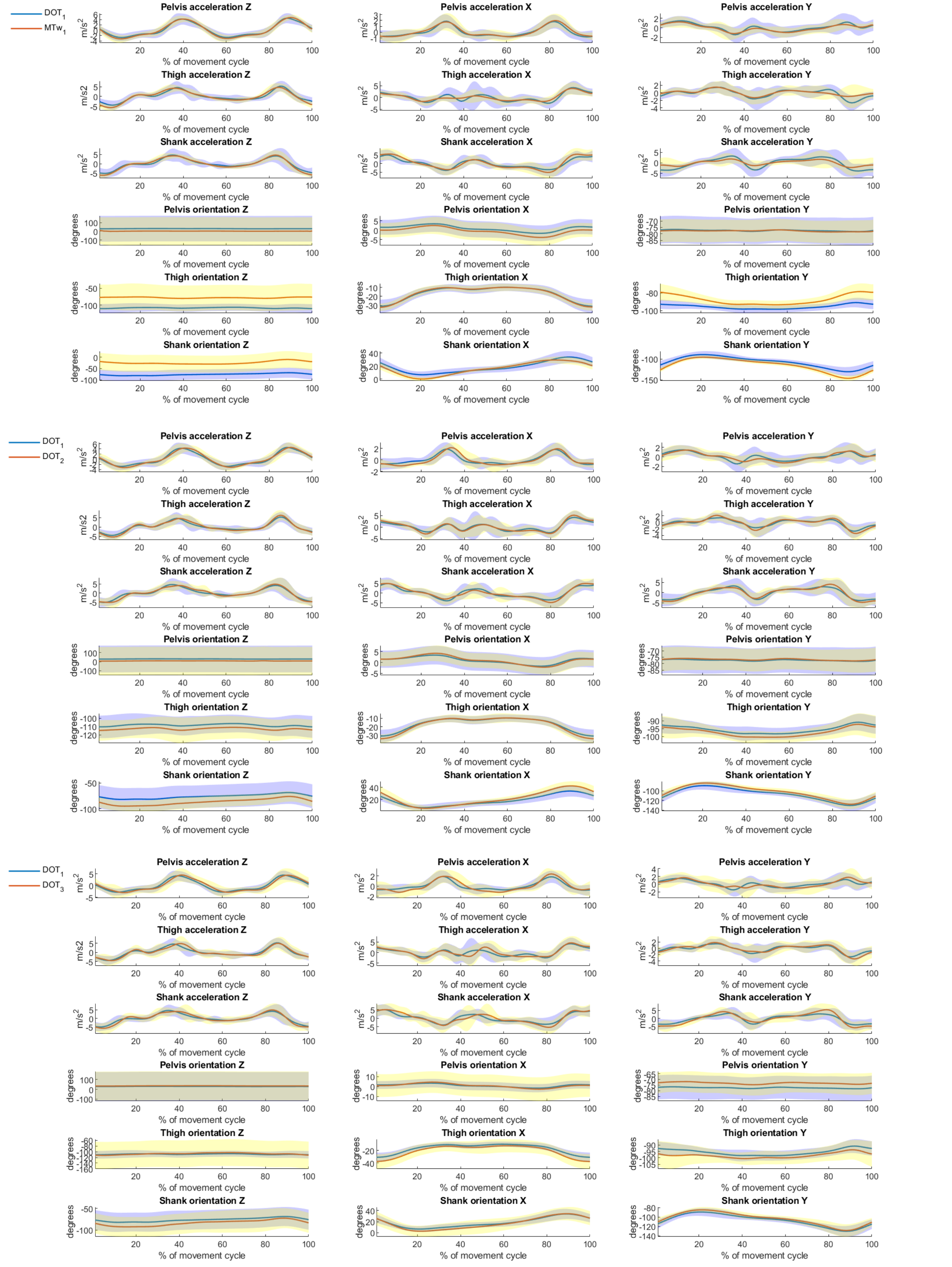


**Supplementary Figure 5.** Mean (stds) waveforms of accelerations and orientations across all participants during stair descents

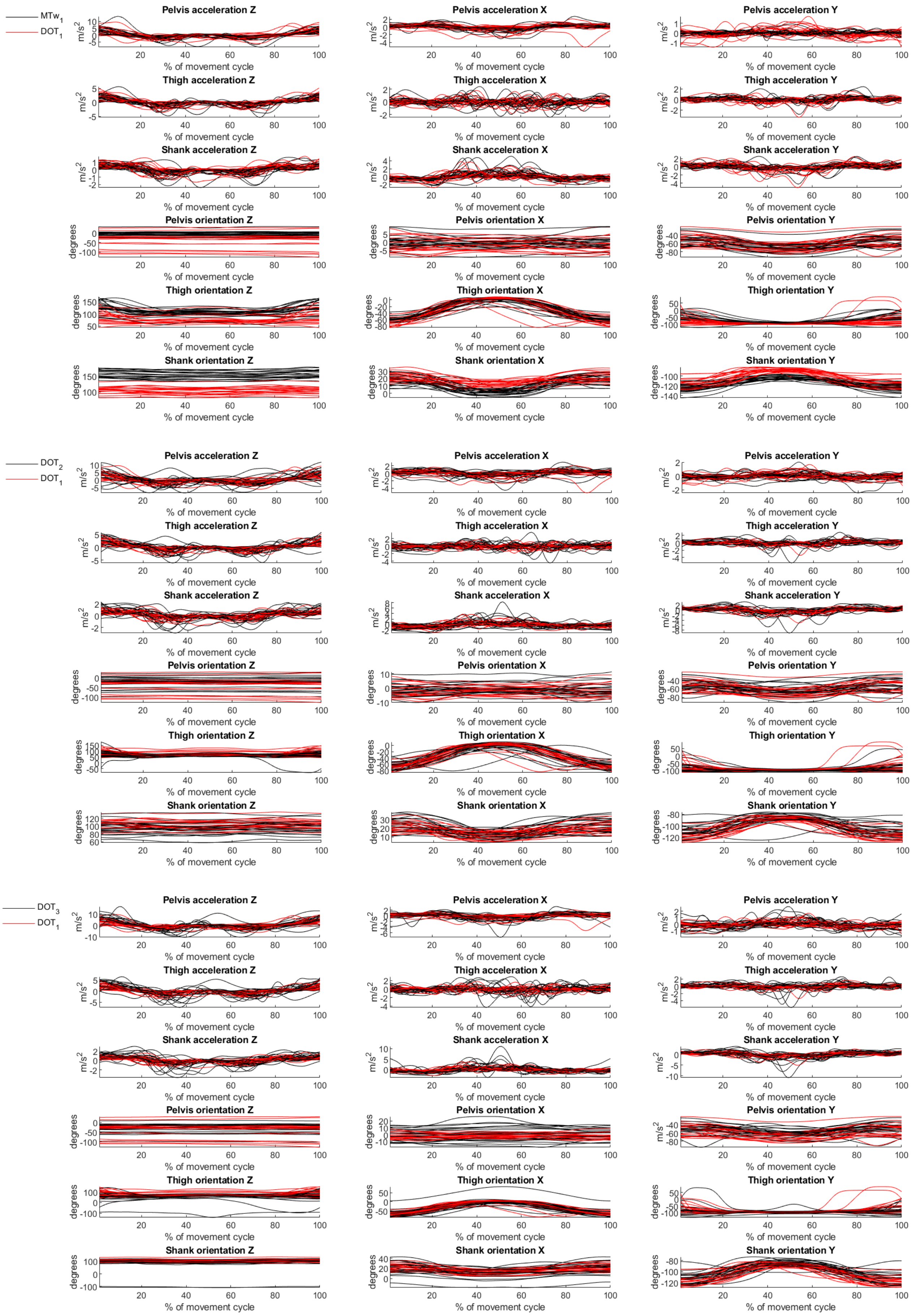


**Supplementary Figure 6**. Mean waveforms of accelerations and orientations for each participant during squats

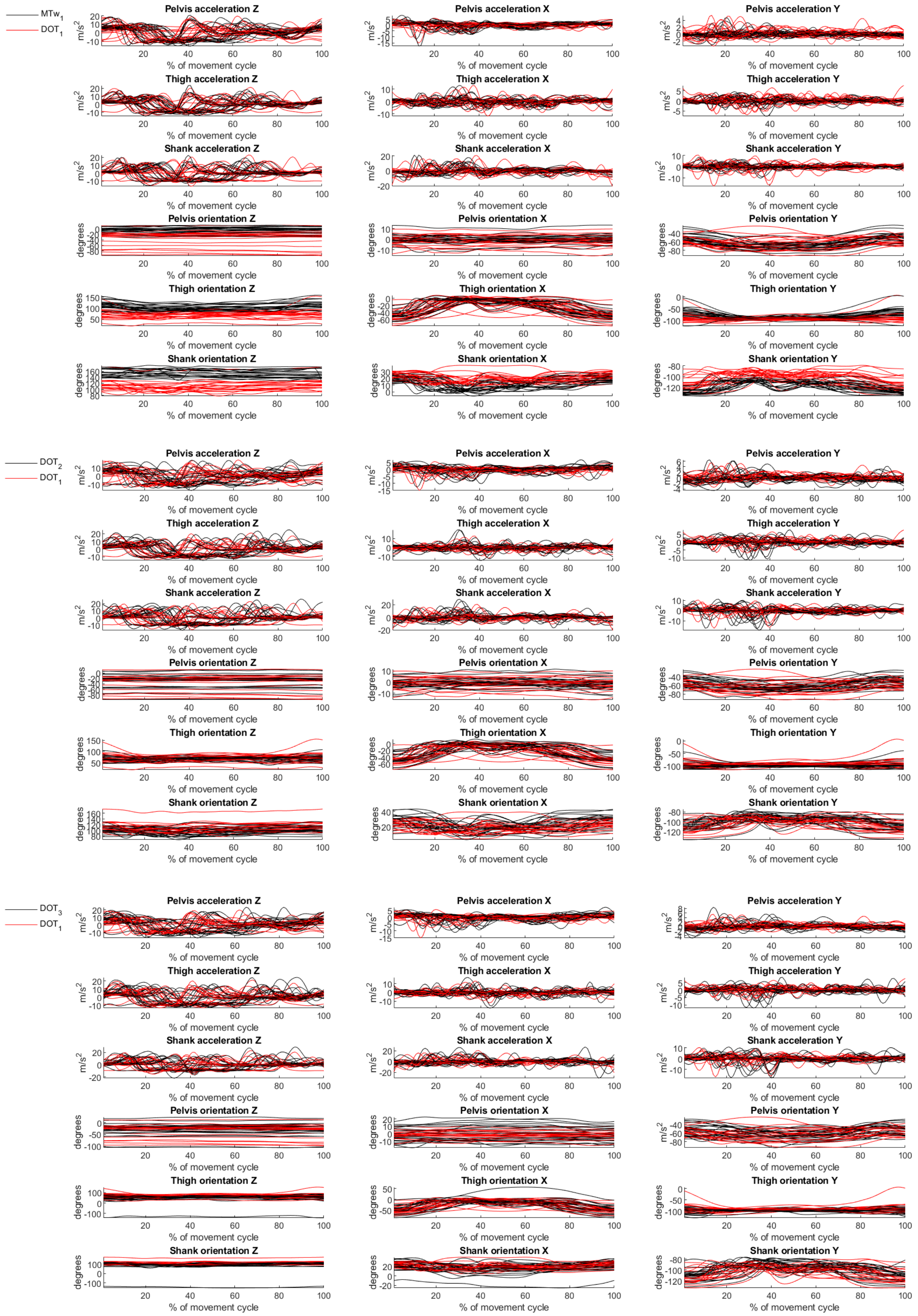


**Supplementary Figure 7**. Mean waveforms of accelerations and orientations for each participant during jumps

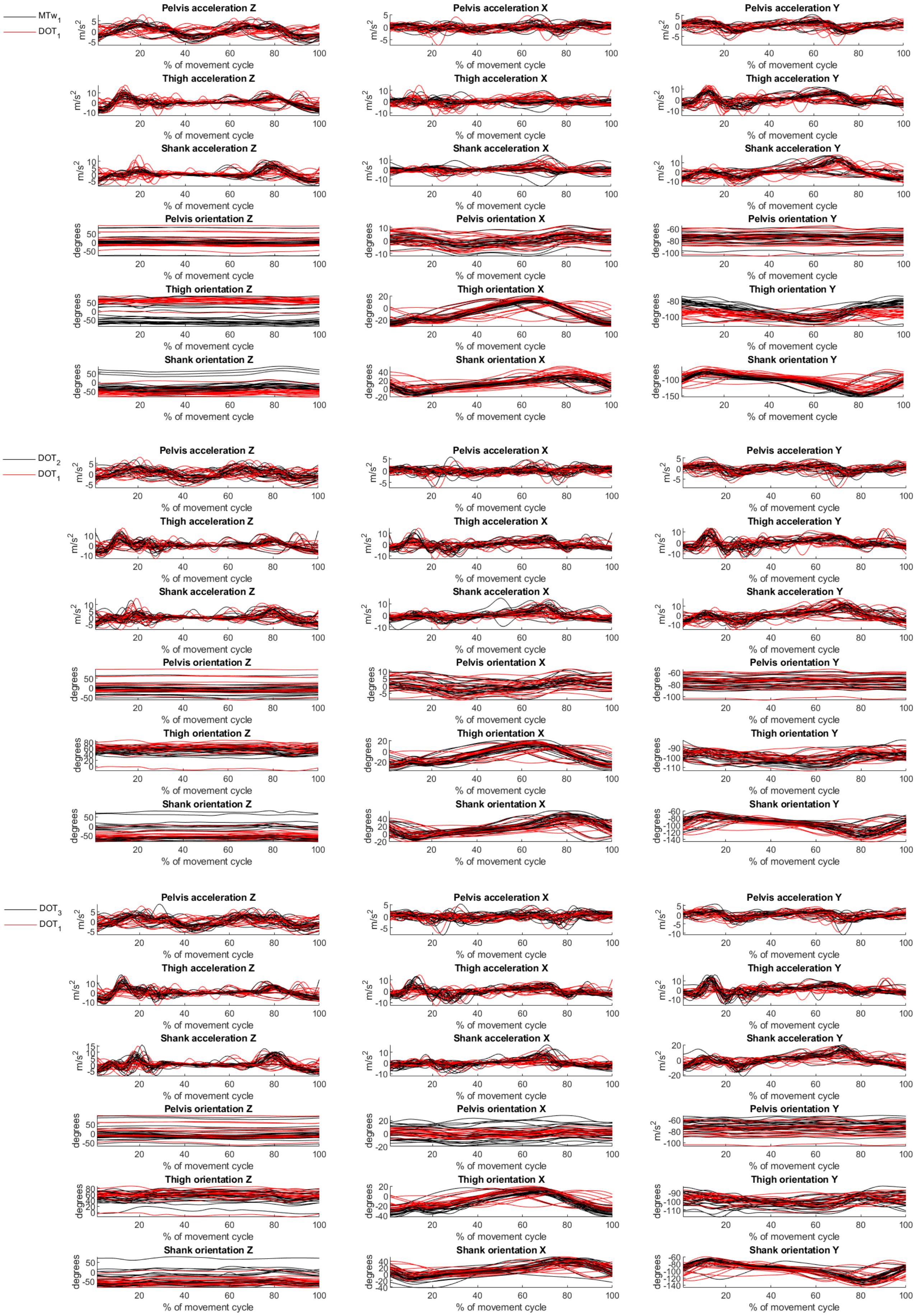

**Supplementary Figure 8**. Mean waveforms of accelerations and orientations for each participant during walking

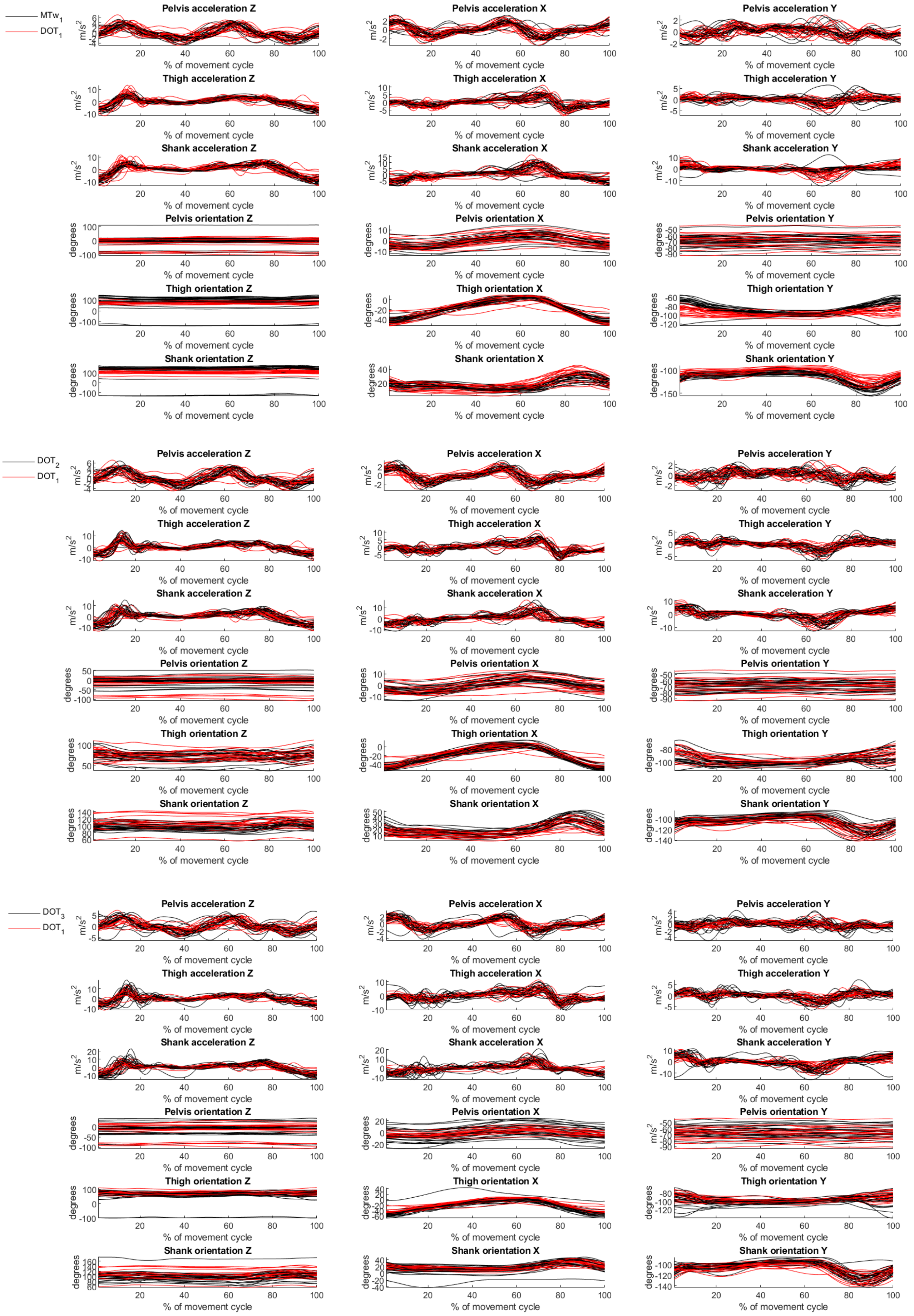


**Supplementary Figure 9**. Mean waveforms of accelerations and orientations for each participant during stair ascents

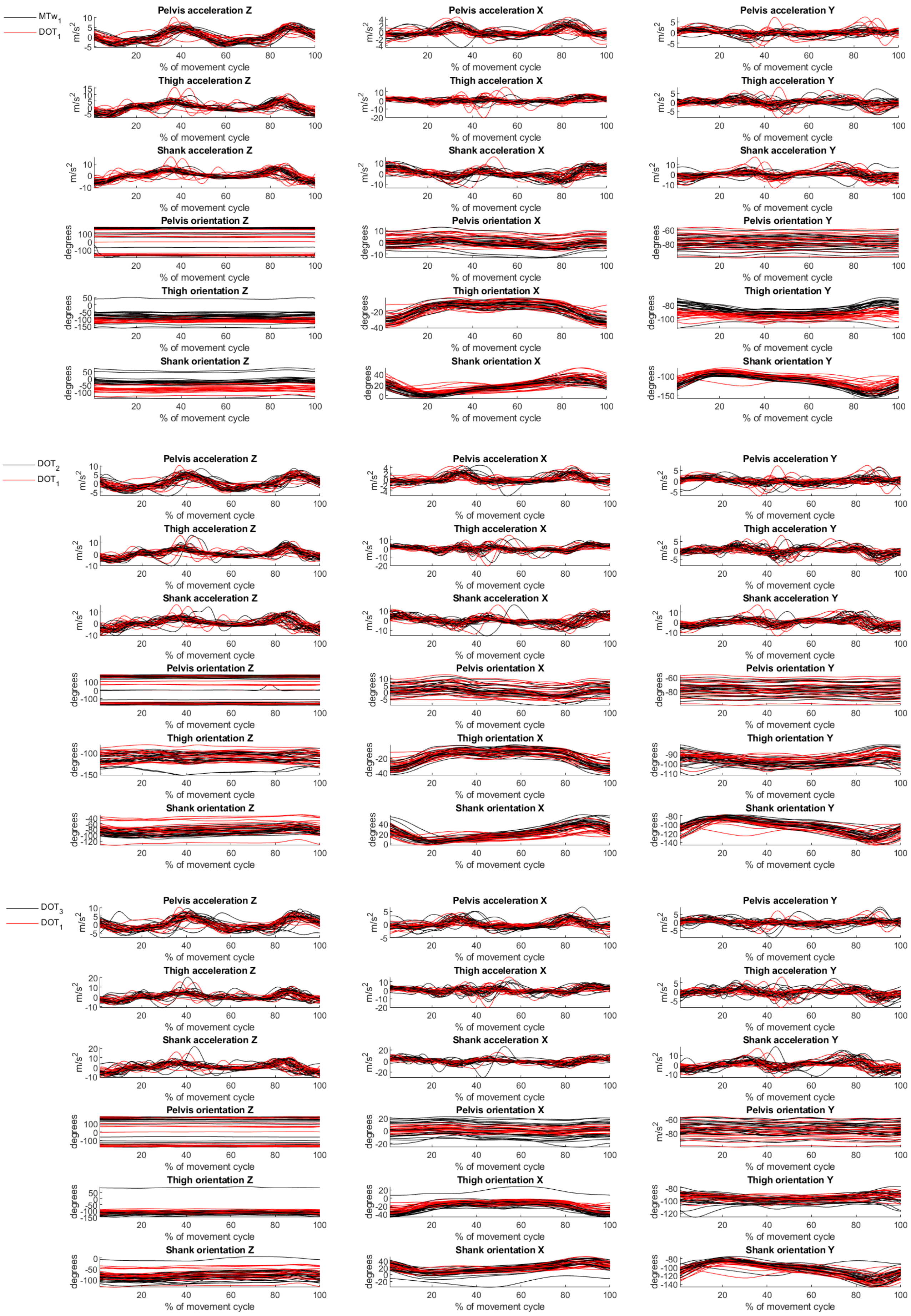


**Supplementary Figure 10**. Mean waveforms of accelerations and orientations for each participant during stair descents

**Supplementary Table 1**. Means [stds] for accelerations (m/s²) and orientations (º) range values collected from the Xsens Dot and Xsens MTw during the first data collection session

| | | Squat | | Jump | | Walk | | Stair ascent | | Stair descent | |
|---|---|---|---|---|---|---|---|---|---|---|---|
| | | DOT | MTw | DOT | MTw | DOT | MTw | DOT | MTw | DOT | MTw |
| *Accelerations* | | | | | | | | | | | |
| Sacrum | *z* | 7.9 [3.9] | 7.6 [ 4.6] | 24.2 [5.2] | 25.8 [4.9] | 7.1 [2.7] | 7.2 [2.2] | 7.1 [1.7] | 7.0 [1.5] | 9.6 [2.7] | 9.1 [1.6] |
| | *x* | 1.9 [1.3] | 2.0 [1.3] | 8.1 [4.5] | 10.1 [4.4] | 4.8 [2.1] | 4.2 [1.5] | 4.0 [1.1] | 3.9 [0.9] | 4.1 [1.7] | 4.3 [1.5] |
| | *y* | 1.2 [0.8] | 0.7 [0.4] | 4.7 [2.1] | 3.3 [1.7] | 6.2 [2.4] | 5.7 [1.8] | 3.3 [0.7] | 2.9 [1.0] | 5.0 [3.4] | 3.8 [1.9] |
| Thigh | *z* | 4.4 [2.2] | 4.1 [2.2] | 25.0 [4.7] | 24.7 [4.9] | 17.3 [6.0] | 16.5 [3.9] | 14.8 [4.7] | 13.0 [2.9] | 13.0 [4.4] | 12.4 [2.7] |
| | *x* | 1.9 [1.0] | 2.1 [1.1] | 11.6 [5.1] | 10.9 [3.7] | 13.3 [5.5] | 7.6 [4.2] | 11.7 [3.4] | 9.7 [3.3] | 12.8 [7.5] | 8.6 [2.7] |
| | *y* | 1.7 [1.2] | 1.7 [1.1] | 8.2 [3.4] | 6.2 [2.0] | 18.9 [6.2] | 14.8 [4.9] | 5.9 [1.9] | 6.0 [3.2] | 7.1 [3.6] | 6.2 [2.8] |
| Shank | *z* | 1.9 [0.9] | 1.7 [0.9] | 25.9 [5.1] | 24.6 [4.9] | 12.0 [4.5] | 12.5 [2.8] | 16.5 [4.8] | 15.1 [2.7] | 15.0 [4.6] | 12.8 [2.1] |
| | *x* | 2.9 [1.6] | 3.3 [2.0] | 21.4 [6.0] | 19.7 [6.0] | 11.8 [5.5] | 10.0 [6.3] | 14.8 [4.4] | 14.8 [4.7] | 14.8 [6.1] | 14.6 [4.6] |
| | *y* | 2.9 [2.0] | 2.1 [1.5] | 14.0 [5.7] | 8.9 [3.6] | 22.6 [6.2] | 20.4 [6.6] | 12.2 [4.8] | 7.5 [5.8] | 13.9 [6.7] | 8.6 [6.0] |
| *Orientations* | | | | | | | | | | | |
| Sacrum | *z* | 6.2 [4.9] | 4.6 [2.0] | 6.6 [3.3] | 5.6 [2.5] | 8.4 [4.8] | 9.3 [4.4] | 7.9 [5.3] | 7.0 [3.3] | 7.3 [4.0] | 8.2 [7.9] |
| | *x* | 3.2 [1.7] | 3.6 [1.4] | 3.7 [2.1] | 3.8 [1.5] | 8.4 [2.9] | 8.4 [2.6] | 10.1 [3.3] | 11.0 [2.4] | 5.9 [2.0] | 7.0 [2.4] |
| | *y* | 20.6 [9.3] | 20.8 [10.2] | 22.6 [5.5] | 24.6 [8.1] | 4.0 [2.0] | 4.4 [1.8] | 3.5 [1.6] | 4.6 [1.6] | 3.8 [1.9] | 3.8 [1.9] |
| Thigh | *z* | 31.1 [25.1] | 33.0 [21.1] | 18.3 [16.2] | 22.0 [12.8] | 15.8 [5.5] | 19.5 [4.6] | 15.6 [6.0] | 18.6 [6.9] | 12.6 [4.9] | 11.1 [5.1] |
| | *x* | 68.4 [12.4] | 59.6 [10.9] | 52.7 [11.8] | 47.8 [9.7] | 39.5 [6.1] | 38.3 [3.5] | 50.0 [9.3] | 47.0 [5.4] | 24.0 [5.4] | 23.7 [3.1] |
| | *y* | 38.0 [33.1] | 54.1 [27.3] | 16.4 [19.6] | 29.9 [18.4] | 13.3 [2.6] | 22.6 [4.0] | 16.1 [6.3] | 31.4 [6.5] | 9.9 [4.0] | 17.3 [4.0] |
| Shank | *z* | 7.5 [2.8] | 8.4 [4.0] | 18.8 [7.6] | 16.9 [11.5] | 17.1 [6.1] | 31.4 [3.7] | 12.6 [4.4] | 23.3 [11.4] | 19.8 [6.4] | 22.5 [4.0] |
| | *x* | 12.3 [6.5] | 19.1 [5.6] | 14.4 [4.5] | 20.1 [4.6] | 47.5 [11.5] | 38.1 [6.0] | 23.1 [6.2] | 18.5 [5.6] | 30.2 [9.3] | 30.0 [5.7] |
| | *y* | 28.7 [7.9] | 22.6 [8.7] | 26.5 [8.5] | 23.6 [5.0] | 57.6 [11.3] | 68.1 [6.1] | 31.4 [8.5] | 37.0 [5.4] | 46.0 [11.6] | 51.7 [7.0] |

**Supplementary Table 2**. Means [stds] for accelerations (m/s²) and orientations (º) range values collected from the Xsens Dot during the first (sensors attached by a researcher) and second (sensors re-attached by the same researcher) data collection sessions

| | | Squat | | Jump | | Walk | | Stair ascent | | Stair descent | |
|---|---|---|---|---|---|---|---|---|---|---|---|
| | | Session 1 | Session 2 | Session 1 | Session 2 | Session 1 | Session 2 | Session 1 | Session 2 | Session 1 | Session 2 |
| *Accelerations* | | | | | | | | | | | |
| Sacrum | *z* | 7.9 [3.9] | 10.2 [4.0] | 24.2 [5.2] | 25.7 [5.2] | 7.1 [2.7] | 6.7 [2.0] | 7.1 [1.7] | 7.6 [1.3] | 9.6 [2.7] | 10.0 [2.4] |
| | *x* | 1.9 [1.3] | 2.7 [1.6] | 8.1 [4.5] | 10.4 [3.9] | 4.8 [2.1] | 4.7 [2.2] | 4.0 [1.1] | 4.3 [1.0] | 4.1 [1.7] | 4.6 [1.6] |
| | *y* | 1.2 [0.8] | 1.2 [0.7] | 4.7 [2.1] | 5.6 [2.4] | 6.2 [2.4] | 6.0 [2.4] | 3.3 [0.7] | 3.5 [1.0] | 5.0 [3.4] | 4.9 [2.3] |
| Thigh | *z* | 4.4 [2.2] | 6.0 [2.5] | 25.0 [4.7] | 26.7 [5.9] | 17.3 [6.0] | 16.5 [5.1] | 14.8 [4.7] | 15.7 [4.1] | 13.0 [4.4] | 14.0 [3.8] |
| | *x* | 1.9 [1.0] | 3.2 [1.6] | 11.6 [5.1] | 13.8 [7.8] | 13.3 [5.5] | 13.8 [4.2] | 11.7 [3.4] | 11.8 [2.7] | 12.8 [7.5] | 12.1 [5.3] |
| | *y* | 1.7 [1.2] | 2.6 [1.8] | 8.2 [3.4] | 10.0 [4.7] | 18.9 [6.2] | 17.0 [4.5] | 5.9 [1.9] | 6.7 [2.7] | 7.1 [3.6] | 7.7 [2.5] |
| Shank | *z* | 1.9 [0.9] | 2.7 [1.3] | 25.9 [5.1] | 28.9 [6.5] | 12.0 [4.5] | 11.8 [3.7] | 16.5 [4.8] | 17.4 [4.4] | 15.0 [4.6] | 15.2 [3.0] |
| | *x* | 2.9 [1.6] | 4.2 [2.1] | 21.4 [6.0] | 22.9 [8.4] | 11.8 [5.5] | 14.5 [4.5] | 14.8 [4.4] | 15.6 [4.0] | 14.8 [6.1] | 15.7 [4.8] |
| | *y* | 2.9 [2.0] | 4.3 [2.3] | 14.0 [5.7] | 16.9 [7.7] | 22.6 [6.2] | 20.0 [5.0] | 12.2 [4.8] | 13.3 [4.0] | 13.9 [6.7] | 14.2 [3.7] |
| *Orientations* | | | | | | | | | | | |
| Sacrum | *z* | 6.2 [4.9] | 4.9 [2.0] | 6.6 [3.3] | 5.6 [2.5] | 8.4 [4.8] | 8.5 [4.7] | 7.9 [5.3] | 6.9 [3.9] | 7.3 [4.0] | 7.3 [3.1] |
| | *x* | 3.2 [1.7] | 3.7 [1.5] | 3.7 [2.1] | 4.5 [2.3] | 8.4 [2.9] | 8.3 [3.1] | 10.1 [3.3] | 11.4 [2.4] | 5.9 [2.0] | 7.0 [3.2] |
| | *y* | 20.6 [9.3] | 20.8 [10.1] | 22.6 [5.5] | 21.9 [9.8] | 4.0 [2.0] | 4.0 [2.0] | 3.5 [1.6] | 4.0 [1.5] | 3.8 [1.9] | 3.7 [2.1] |
| Thigh | *z* | 31.1 [25.1] | 26.0 [19.2] | 18.3 [16.2] | 16.5 [7.6] | 15.8 [5.5] | 15.8 [5.6] | 15.6 [6.0] | 16.7 [6.0] | 12.6 [4.9] | 11.5 [5.5] |
| | *x* | 68.4 [12.4] | 68.1 [11.5] | 52.7 [11.8] | 49.3 [14.3] | 39.5 [6.1] | 40.5 [3.7] | 50.0 [9.3] | 53.6 [3.5] | 24.0 [5.4] | 25.7 [3.5] |
| | *y* | 38.0 [33.1] | 29.4 [28.7] | 16.4 [19.6] | 12.2 [10.5] | 13.3 [2.6] | 13.0 [2.9] | 16.1 [6.3] | 16.0 [6.8] | 9.9 [4.0] | 10.3 [3.5] |
| Shank | *z* | 7.5 [2.8] | 8.7 [2.4] | 18.8 [7.6] | 19.2 [7.4] | 17.1 [6.1] | 17.9 [6.2] | 12.6 [4.4] | 14.6 [6.6] | 19.8 [6.4] | 22.8 [6.8] |
| | *x* | 12.3 [6.5] | 14.1 [6.5] | 14.4 [4.5] | 16.5 [5.8] | 47.5 [11.5] | 54.6 [9.7] | 23.1 [6.2] | 27.7 [4.6] | 30.2 [9.3] | 31.9 [7.2] |
| | *y* | 28.7 [7.9] | 26.5 [8.6] | 26.5 [8.5] | 24.7 [10] | 57.6 [11.3] | 51.2 [12.8] | 31.4 [8.5] | 31.1 [7.9] | 46.0 [11.6] | 46.5 [8.7] |

**Supplementary Table 3**. Means [stds] for accelerations (m/s²) and orientations (º) range values collected from the Xsens Dot during the first (sensors attached by a researcher) and third (sensors attached by participants) data collection sessions

| | | **Squat** | | **Jump** | | **Walk** | | **Stair ascent** | | **Stair descent** | |
|---|---|---|---|---|---|---|---|---|---|---|---|
| | | Session 1 | Session 3 | Session 1 | Session 3 | Session 1 | Session 3 | Session 1 | Session 3 | Session 1 | Session 3 |
| *Accelerations* | | | | | | | | | | | |
| Sacrum | *z* | 7.9 [3.9] | 12.3 [4.8] | 24.2 [5.2] | 28.1 [5.6] | 7.1 [2.7] | 7.9 [2.4] | 7.1 [1.7] | 8.5 [2.3] | 9.6 [2.7] | 11.1 [2.8] |
| | *x* | 1.9 [1.3] | 3.5 [2.1] | 8.1 [4.5] | 10.3 [3.3] | 4.8 [2.1] | 5.9 [2.3] | 4.0 [1.1] | 5.0 [1.1] | 4.1 [1.7] | 5.9 [2.0] |
| | *y* | 1.2 [0.8] | 1.9 [0.9] | 4.7 [2.1] | 5.9 [2.5] | 6.2 [2.4] | 6.6 [2.6] | 3.3 [0.7] | 4.5 [1.6] | 5.0 [3.4] | 6.8 [4.1] |
| Thigh | *z* | 4.4 [2.2] | 7.6 [3.0] | 25.0 [4.7] | 26.3 [5.4] | 17.3 [6.0] | 18.2 [6.0] | 14.8 [4.7] | 18.7 [5.6] | 13.0 [4.4] | 14.4 [4.8] |
| | *x* | 1.9 [1.0] | 3.4 [1.7] | 11.6 [5.1] | 14.3 [7.1] | 13.3 [5.5] | 14.7 [4.6] | 11.7 [3.4] | 13.4 [3.0] | 12.8 [7.5] | 13.4 [5.2] |
| | *y* | 1.7 [1.2] | 2.8 [1.9] | 8.2 [3.4] | 10.7 [5.0] | 18.9 [6.2] | 22.0 [6.8] | 5.9 [1.9] | 7.3 [2.4] | 7.1 [3.6] | 8.6 [3.1] |
| Shank | *z* | 1.9 [0.9] | 3.1 [1.5] | 25.9 [5.1] | 28.1 [7.1] | 12.0 [4.5] | 14.7 [4.1] | 16.5 [4.8] | 21.8 [6.2] | 15.0 [4.6] | 16.2 [3.2] |
| | *x* | 2.9 [1.6] | 5.1 [2.9] | 21.4 [6.0] | 25.1 [10.1] | 11.8 [5.5] | 14.4 [4.9] | 14.8 [4.4] | 19.4 [5.4] | 14.8 [6.1] | 19.2 [10.2] |
| | *y* | 2.9 [2.0] | 4.9 [3.1] | 14.0 [5.7] | 17.5 [6.8] | 22.6 [6.2] | 26.9 [5.8] | 12.2 [4.8] | 16.3 [4.6] | 13.9 [6.7] | 16.9 [6.0] |
| *Orientations* | | | | | | | | | | | |
| Sacrum | *z* | 6.2 [4.9] | 5.6 [3.3] | 6.6 [3.3] | 6.6 [3.2] | 8.4 [4.8] | 13.9 [6.3] | 7.9 [5.3] | 8.3 [3.8] | 7.3 [4.0] | 7.6 [3.5] |
| | *x* | 3.2 [1.7] | 5.0 [3.6] | 3.7 [2.1] | 5.3 [3.3] | 8.4 [2.9] | 11.0 [2.8] | 10.1 [3.3] | 13.7 [4.3] | 5.9 [2.0] | 8.0 [3.5] |
| | *y* | 20.6 [9.3] | 18.5 [8.3] | 22.6 [5.5] | 20.8 [7.4] | 4.0 [2.0] | 6.0 [3.1] | 3.5 [1.6] | 5.3 [2.1] | 3.8 [1.9] | 5.0 [1.9] |
| Thigh | *z* | 31.1 [25.1] | 34.1 [22.0] | 18.3 [16.2] | 20.4 [5.6] | 15.8 [5.5] | 19.0 [6.2] | 15.6 [6.0] | 20.2 [8.2] | 12.6 [4.9] | 16.7 [8.1] |
| | *x* | 68.4 [12.4] | 68.0 [11.5] | 52.7 [11.8] | 50.0 [14.7] | 39.5 [6.1] | 43.1 [4.1] | 50.0 [9.3] | 53.7 [5.2] | 24.0 [5.4] | 27.2 [4.1] |
| | *y* | 38.0 [33.1] | 37.1 [30.7] | 16.4 [19.6] | 13.7 [7.0] | 13.3 [2.6] | 14.9 [4.3] | 16.1 [6.3] | 19.4 [7.4] | 9.9 [4.0] | 11.4 [4.1] |
| Shank | *z* | 7.5 [2.8] | 12.5 [5.0] | 18.8 [7.6] | 24.0 [9.0] | 17.1 [6.1] | 22.5 [8.4] | 12.6 [4.4] | 19.6 [8.0] | 19.8 [6.4] | 25.1 [6.9] |
| | *x* | 12.3 [6.5] | 12.5 [7.1] | 14.4 [4.5] | 13.7 [4.8] | 47.5 [11.5] | 54.5 [7.1] | 23.1 [6.2] | 25.2 [4.7] | 30.2 [9.3] | 34.0 [8.2] |
| | *y* | 28.7 [7.9] | 32.0 [8.0] | 26.5 [8.5] | 29.4 [9.4] | 57.6 [11.3] | 57.9 [8.8] | 31.4 [8.5] | 35.2 [6.0] | 46.0 [11.6] | 48.7 [8.5] |

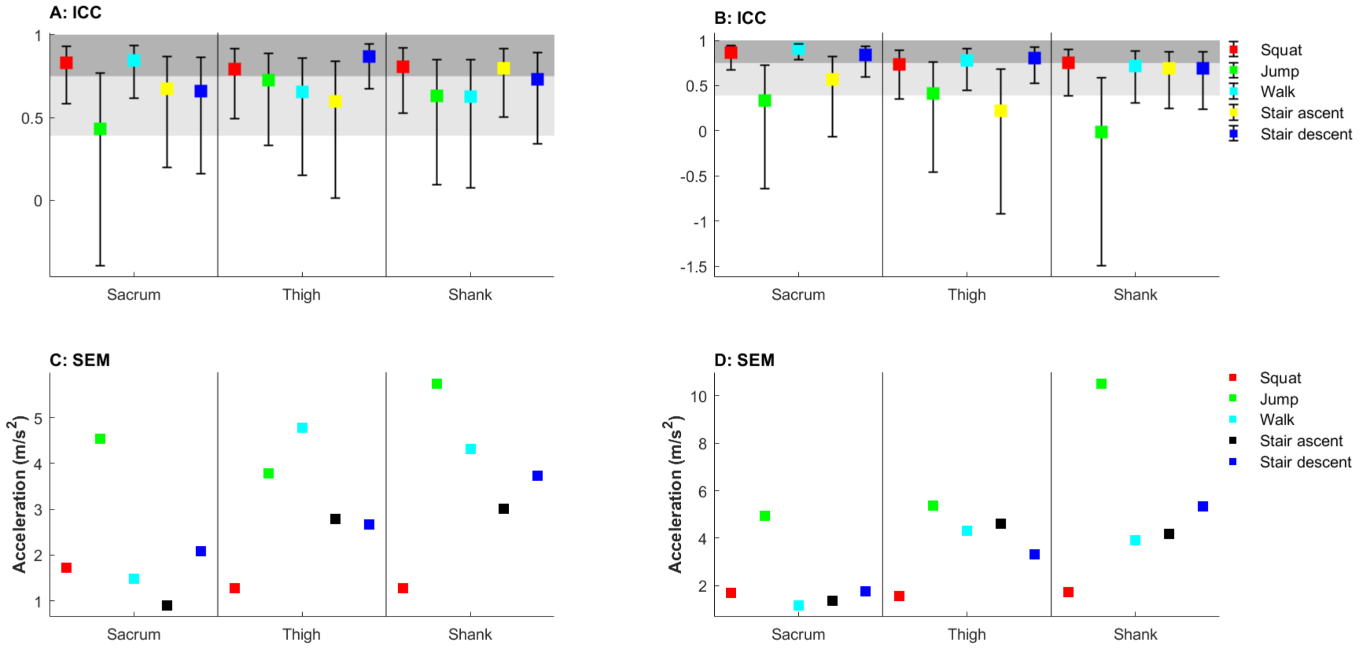


**Supplementary Figure 11**. A. ICCs (95% CI) for Euclidean norm of acceleration range values (test-retest reliability when the Xsens Dot were attached by the researcher; session 1 vs session 2); B. ICCs (95% CI) for acceleration norm range values (test-retest reliability when the Xsens Dot were attached by participants; session 1 vs session 3); light grey shaded areas indicate fair-to-high test-retest reliability; dark grey shaded areas indicate excellent test-retest reliability; C. SEMs of acceleration norm range values (test-retest reliability when the Xsens Dot were attached by the researcher; session 1 vs session 2); D. SEMs of acceleration norm range values (test-retest reliability when the Xsens Dot were attached by participants; session 1 vs session 3); the Euclidean norm of acceleration was quantified using the following equation: acc_norm = sqrt((accX * accX) + (accY * accY) + (accZ * accZ))

**Supplementary Table 4**. Paired-sample t-test comparing estimated load from instrumented pressure insoles between left and right foot during data collection 1 and 2 for squats, vertical jumps, walking, stair ascents, and stair descents (n=17).

| Activity | Mean | Std. deviation | Std. Error Mean | 95% CI LB | 95% CI UB | t | df | Sig. (2-tailed) |
|---|---|---|---|---|---|---|---|---|
| l_sq_1–r_sq_1 | 1.81 | 8.91 | 2.16 | -2.77 | 6.40 | .84 | 16.00 | .41 |
| l_sq_2–r_sq_2 | -1.10 | 8.14 | 1.97 | -5.28 | 3.08 | -.56 | 16.00 | .59 |
| l_vj_1–r_vj_1 | -2.63 | 5.46 | 1.32 | -5.44 | .18 | -1.98 | 16.00 | .06 |
| l_vj_2–r_vj_2 | -1.91 | 6.67 | 1.62 | -5.34 | 1.52 | -1.18 | 16.00 | .26 |
| l_walk_1-r_walk_1 | 2.26 | 4.54 | 1.10 | -.07 | 4.60 | 2.06 | 16.00 | .06 |
| l_walk_2-r_walk_2 | .97 | 5.67 | 1.37 | -1.94 | 3.89 | .71 | 16.00 | .49 |
| l_stair_a_1-r_stair_a_1 | 1.89 | 4.83 | 1.17 | -.59 | 4.38 | 1.62 | 16.00 | .13 |
| l_stair_a_2- r_stair_a_2 | 1.57 | 6.09 | 1.48 | -1.56 | 4.71 | 1.06 | 16.00 | .30 |
| l_stair_d_1-r_stair_d_1 | .84 | 6.09 | 1.48 | -2.30 | 3.97 | .57 | 16.00 | .58 |
| l_stair_d_2-r_stair_d_2 | -.37 | 6.08 | 1.47 | -3.49 | 2.76 | -.25 | 16.00 | .81 |

Abbreviations: l – left; r- right; 1 – first data collection; 2 – second data collection; sq – squat trial; vj – vertical jump trial; walk – walking trial; stair_a – stair ascent trial; stair_d – stair descent trial.
